# Artificial Intelligence and Multi-Omics Approaches in the Precision Management of Pulmonary Hypertension: From Early Diagnosis to Therapeutic Stratification

**DOI:** 10.3390/ijms27177763

**Published:** 2026-08-30

**Authors:** Sergio Ferrantelli, Alessandro Del Cuore, Giuliano Cassataro, Luigi Dell’Ajra, Rosario Norrito, Giulio Geraci, Gabriella Carmina, Chiara Minà, Vincenzo Polizzi, Nicola Ciancio, Carlo Domenico Maida

**Affiliations:** 1 Molecular and Clinical Medicine PhD Program, University of Palermo, 90127 Palermo, Italy; ferrantelli.sergio@gmail.com; 2 Cardiology Unit, San Vito e Santo Spirito Hospital, 91011 Alcamo, Italy; 3 Cardiology Unit, Cervello Hospital, A.O. Ospedali Riuniti Villa Sofia-Cervello, 90146 Palermo, Italy; alex.delcuore@gmail.com (A.D.C.); m.carmina@villasofia.it (G.C.); cmina.anna@gmail.com (C.M.); v.polizzi@villasofia.it (V.P.); 4 Medicine Unit, Fondazione G. Giglio, 90015 Cefalù, Italy; giuliano.cassat@gmail.com; 5 Department of Internal Medicine, S. Elia Hospital, 93100 Caltanissetta, Italy; mail.luigi17.cl@gmail.com; 6 Department of Internal Medicine, Buccheri La Ferla Hospital, 90123 Palermo, Italy; rosario94.norrito@gmail.com; 7 Department of Medicine and Surgery, “Kore” University of Enna, 94100 Enna, Italy; giulio.geraci@unikore.it; 8 Department of Pulmonary Medicine, S. Elia Hospital, 93100 Caltanissetta, Italy; nicola.ciancio@asp.cl.it

**Keywords:** pulmonary hypertension, pulmonary arterial hypertension, artificial intelligence, machine learning, multi-omics, molecular endotyping, right-ventricular phenotyping, precision medicine, risk stratification, therapeutic stratification

## Abstract

Pulmonary hypertension (PH) is a heterogeneous clinical syndrome in which similar haemodynamic abnormalities may arise from distinct vascular, cardiac, pulmonary, thromboembolic, and molecular mechanisms. This complexity limits the ability of conventional classifications and risk scores to fully capture individual disease trajectories and treatment responses. Artificial intelligence (AI) offers a framework for integrating clinical data, electrocardiography, multimodal imaging, invasive haemodynamics, biomarkers, and multi-omics information across the PH care pathway. This review summarises current applications of machine learning and deep learning in early detection, diagnostic referral, right-ventricular and pulmonary vascular phenotyping, molecular endotyping, risk stratification, and therapeutic decision support. Available studies show promising results for AI-assisted electrocardiographic screening, automated echocardiographic and cardiac magnetic resonance analysis, computed tomography (CT)-based phenotyping, and multimodal prognostic modelling. True multi-omics integration in PH remains limited to discovery studies and has not yet yielded externally validated endotype or treatment-response classifiers. Evidence maturity is task-dependent: screening and phenotyping span several PH groups, whereas validated risk tools, molecular endotyping, and pathway-directed therapy remain predominantly PAH-based, particularly in idiopathic/heritable PAH. However, most evidence remains retrospective, derives from selected referral populations, and lacks robust external or prospective validation. No AI-based model currently supports routine drug selection or autonomous clinical decision-making. Future progress will require harmonised multicentre datasets and standardised acquisition protocols, transparent and interpretable models, and prospective studies demonstrating meaningful clinical benefit. AI should therefore be viewed as an emerging decision-support tool that may strengthen precision medicine in PH while complementing clinical expertise across diagnosis, phenotyping, risk assessment, and therapeutic stratification pathways.

## 1. Introduction

Pulmonary hypertension (PH) is simultaneously a haemodynamic abnormality, a family of mechanistically distinct diseases, and a common complication of prevalent cardiac and pulmonary disorders [1,2,3]. It represents a haemodynamic final common pathway produced by divergent vascular, cardiac, pulmonary, thromboembolic, and systemic disorders [2,3]. This mismatch—one pressure abnormality arising from multiple biological causes—makes PH an archetypal precision-medicine problem: the task is not merely to detect elevated pressure, but to identify the mechanism, phenotype, and trajectory that matter for the individual patient [3,4]. The contemporary definition has moved the diagnostic boundary closer to the upper limit of normal: PH is present when mean pulmonary arterial pressure (mPAP) is >20 mmHg at rest on right-heart catheterisation [1,5,6]. Pre-capillary PH requires pulmonary arterial wedge pressure (PAWP) ≤ 15 mmHg and pulmonary vascular resistance (PVR) > 2 Wood units (WU); isolated post-capillary PH is defined by PAWP > 15 mmHg and PVR ≤ 2 WU, and combined post- and pre-capillary PH by PAWP > 15 mmHg and PVR > 2 WU [1,5]. Exercise PH is defined by an mPAP/cardiac-output slope > 3 mmHg/L/min from rest to exercise [1]. These haemodynamic categories sit alongside the five-group clinical classification: pulmonary arterial hypertension (PAH; group 1), PH associated with left-heart disease (group 2), lung disease and/or hypoxia (group 3), pulmonary artery obstruction, principally chronic thromboembolic disease (group 4), and unclear or multifactorial mechanisms (group 5) [1,2,5]. The classification remains clinically useful, but its borders are increasingly porous in older, multimorbid patients [3,4].

The scale of the problem is easily underestimated because PAH—the best-characterised therapeutic subgroup—is rare, whereas PH as a whole is not [2]. PH affects at least 1% of the global population, with groups 2 and 3 accounting for most cases and prevalence rising sharply with age [2,7]. Registry data place PAH incidence at approximately 2.5–7.5 cases per million per year and prevalence at 15–50 per million, although estimates vary with ascertainment [7,8]. In interstitial lung disease, a large meta-analysis found a pooled PH prevalence of 36% by right-heart catheterisation and 34% by echocardiography; PH associated with interstitial lung disease was linked to greater symptom burden and worse prognosis [9,10]. Across aetiologies, progressive pulmonary vascular load drives right-ventricular (RV) remodelling and failure, a major proximate cause of death, while repeated investigations, specialist care, hospitalisation, and advanced therapies generate a burden that increases with severity [2,11]. Yet patients often enter this pathway late. In the REVEAL registry, the mean interval from symptom onset to diagnostic catheterisation was 2.8 years, reflecting non-specific dyspnoea, fragmented testing, and delayed specialist recognition [12]. Current recommendations therefore endorse fast-track referral to a PH centre whenever severe pulmonary vascular disease or right-heart failure is suspected [1,6]. Lower haemodynamic thresholds may expose disease earlier, but they also sharpen the phenotyping problem: in EVIDENCE-PAH UK, most patients with mPAP of 21–24 mmHg had underlying heart or lung disease, and mildly abnormal mPAP or PVR predicted poorer survival [13].

**Figure 1:**
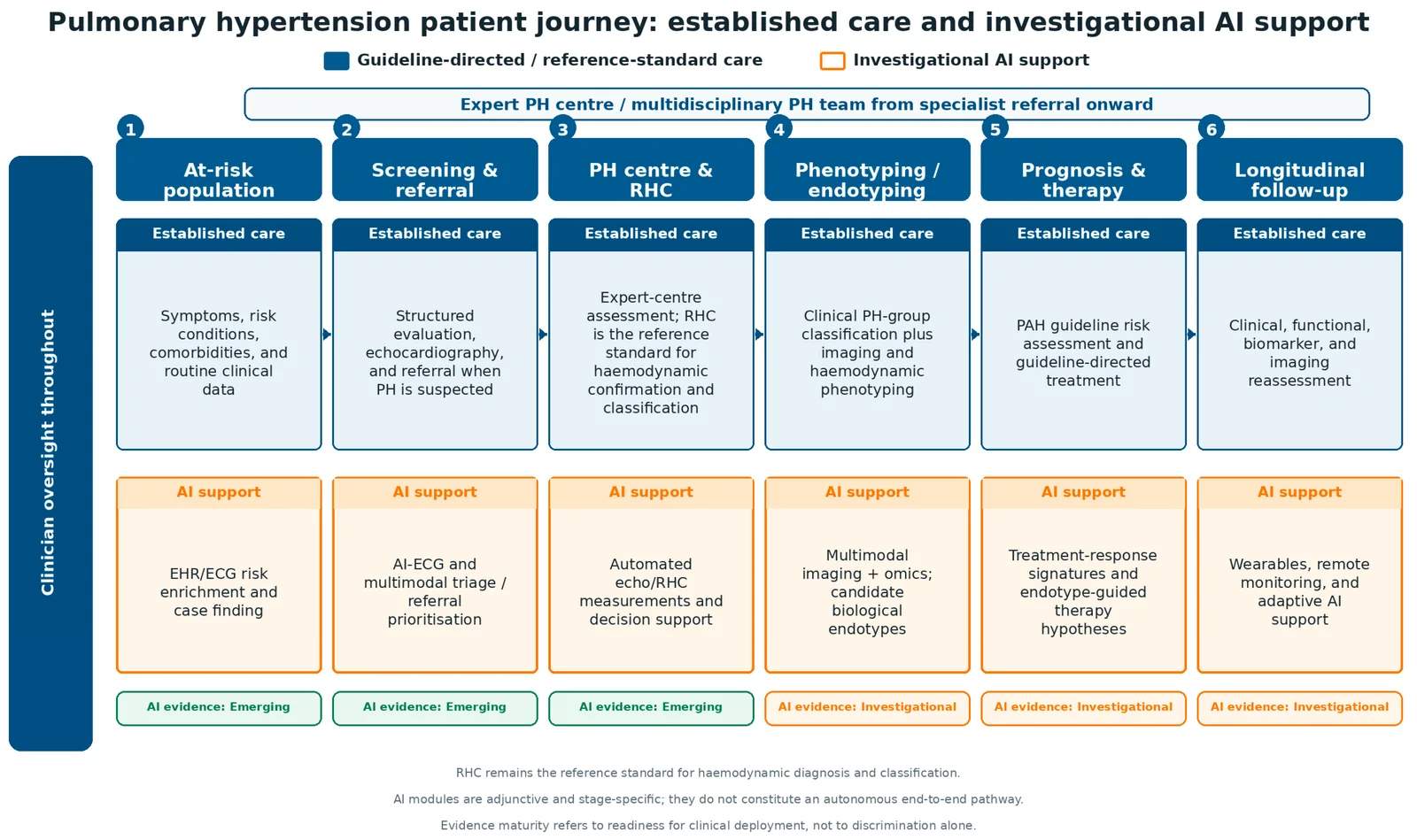
Established clinical pathway and investigational AI support across the pulmonary hypertension patient journey. Solid blue boxes and arrows denote guideline-directed or reference-standard clinical steps, whereas dashed orange boxes denote investigational AI support. Right-heart catheterisation (RHC) at an expert PH centre remains the reference standard for haemodynamic confirmation and classification. AI evidence maturity is shown qualitatively at each stage and does not imply clinical approval or readiness for autonomous use. Clinician oversight applies throughout. This figure was created with the assistance of artificial intelligence and subsequently reviewed and curated by the authors.

Validated PAH risk scores combining functional class, exercise capacity, biomarkers, and haemodynamics provide an established prognostic function within their intended PAH populations [3,4,5]. Their purpose is not to resolve differential diagnosis across PH groups or to identify molecular endotypes, and these separate tasks should not be interpreted as shortcomings of validated prognostic tools. Differential phenotyping is needed to distinguish overlapping or mixed cardiopulmonary presentations, including group 2/3-like phenotypes, whereas molecular endotyping seeks biologically coherent mechanisms that may underlie heterogeneity within clinically defined disease. Artificial intelligence (AI) may complement, rather than replace, conventional risk assessment by integrating longitudinal electronic health records, electrocardiography (ECG), imaging, invasive haemodynamics, biomarkers, genomics, and other omics for these distinct objectives [4,14]. An NHS England model used pre-diagnostic healthcare-use patterns from 709 patients with idiopathic PAH and more than 2.8 million controls to identify a disease-enriched population, although its low sensitivity and cross-validation-only design preclude clinical deployment [15]. In 256 newly diagnosed patients with PH, machine learning of three-dimensional cardiac magnetic resonance (CMR) motion improved one-year survival discrimination beyond conventional markers [16]. Unsupervised analysis of circulating immune proteins identified four PAH clusters with distinct survival, illustrating how omics can identify biologically informative molecular phenotypes that may be evaluated as candidate endotypes rather than assumed to represent distinct mechanisms [17]. The current evidence nevertheless remains predominantly retrospective, single-centre, or internally validated. The central objective is therefore to convert pattern recognition into externally validated, calibrated, explainable, and prospectively tested tools that shorten referral, deepen phenotyping, and improve clinical decisions without amplifying bias. Figure 1 distinguishes established guideline-directed and reference-standard clinical steps from investigational AI support across the PH care pathway.

**Figure 2:**
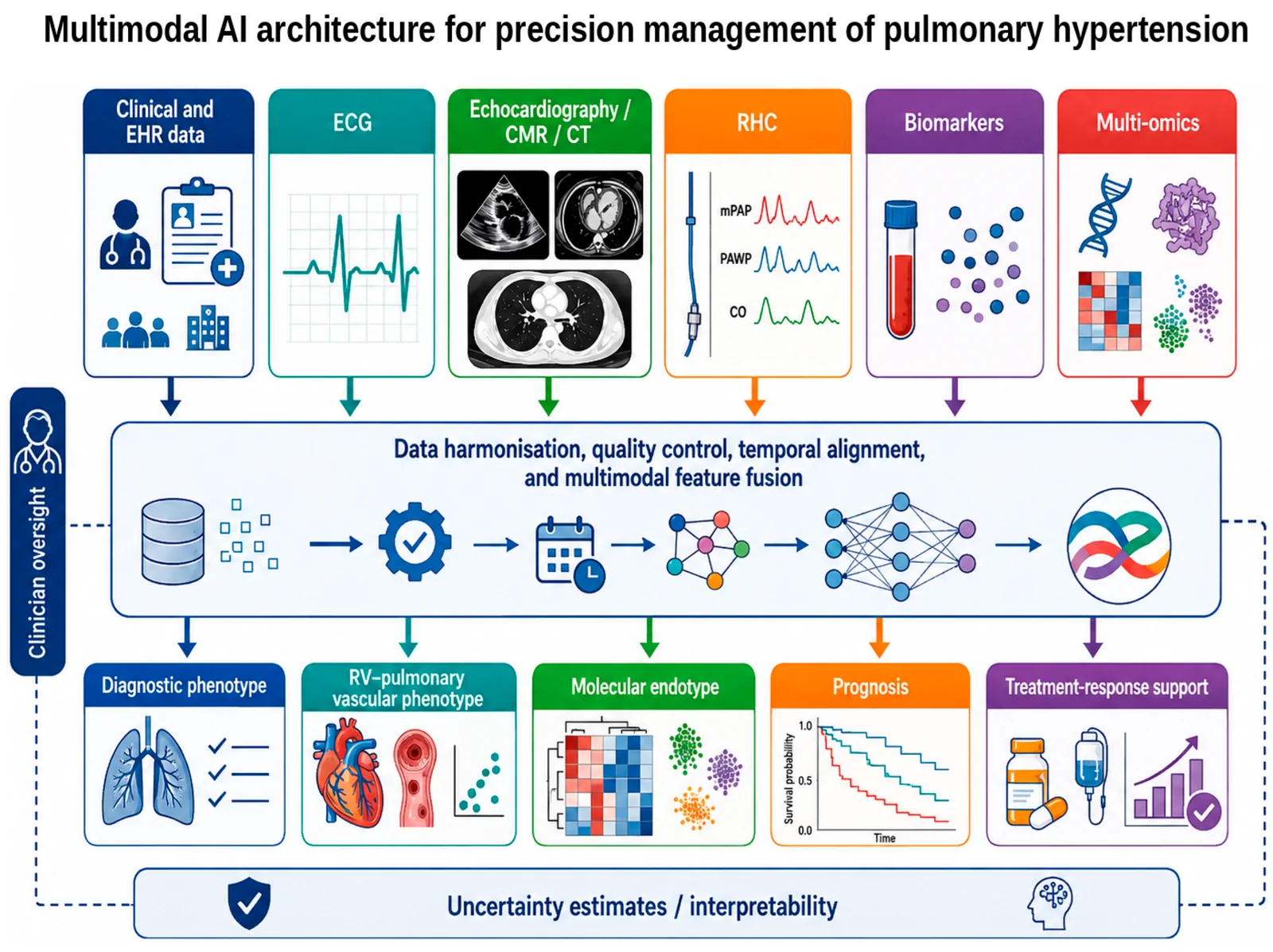
Multimodal AI architecture for precision management of pulmonary hypertension. The schematic shows how heterogeneous clinical, physiological, imaging, and molecular inputs are harmonised and fused into interpretable outputs for diagnosis, phenotyping, prognosis, and therapeutic stratification, with uncertainty estimates and clinician oversight. This figure was created with the assistance of artificial intelligence and subsequently reviewed and curated by the authors. The figure operationalises the molecular pathophysiology-to-therapeutic stratification sequence by linking cross-modal phenotypes to candidate biological endotypes, prognosis, and treatment-response support. This schematic represents a proposed translational architecture; it does not imply that a clinically validated PH model currently integrates all depicted molecular, imaging, haemodynamic, and clinical layers.

Throughout this review, we therefore adopt a sequential translational framework in which molecular pathophysiology gives rise to measurable electrical, imaging, haemodynamic, circulating, and molecular phenotypes; AI integrates these partially overlapping signals to identify clinically meaningful phenotypes and candidate biological endotypes; mechanistically validated endotypes may then inform prognosis, therapeutic stratification, and biomarker-guided trial enrichment [3,4,14,17]. This sequence is conceptual rather than deterministic: ECG or imaging signatures should not be interpreted as direct surrogates of a molecular pathway without cross-modal and experimental validation. The framework connects the patient journey in Figure 1 with the multimodal architecture presented in Figure 2.

Relative to recent comprehensive and systematic reviews that primarily catalogue AI applications in PH [18,19], the present review integrates AI with molecular pathophysiology and multi-omics, distinguishes observable phenotypes from biologically supported candidate endotypes, and traces the evidentiary pathway from detection to clinically actionable translation. The structure follows the intended use: Section 3 addresses screening and referral; Section 4, diagnostic support and cardiopulmonary phenotyping; Section 5, molecular mechanisms, omics layers, and candidate-endotype validation; Section 6, prognosis; Section 7, prediction of treatment benefit, response monitoring, and trial enrichment; Section 8, cross-cutting methodological, implementation, and regulatory standards; and Section 9, emerging research architectures.

### Literature-Search Methodology

This article was designed as a narrative rather than a systematic review. A structured search of PubMed/MEDLINE was conducted from database inception to 21 August 2026, with the final search performed on that date. The strategy combined the terms “pulmonary hypertension” or “pulmonary arterial hypertension” with terms related to artificial intelligence and machine learning, including “artificial intelligence”, “machine learning”, “deep learning”, and “neural network”; clinical and imaging modalities, including “electrocardiography”, “echocardiography”, “cardiac magnetic resonance”, “computed tomography”, “radiomics”, and “haemodynamics”; and molecular or translational domains, including “biomarker”, “genomics”, “transcriptomics”, “proteomics”, “metabolomics”, “multi-omics”, “non-coding RNA”, “single-cell”, “spatial omics”, “risk stratification”, “treatment response”, “precision medicine”, and “digital twin”. Reference lists of pertinent guidelines, consensus documents, reviews, and primary studies were also screened to identify additional reports.

The exact PubMed/MEDLINE search string was: ((“pulmonary hypertension”[Title/Abstract] OR “pulmonary arterial hypertension”[Title/Abstract]) AND (“artificial intelligence”[Title/Abstract] OR “machine learning”[Title/Abstract] OR “deep learning”[Title/Abstract] OR “neural network”[Title/Abstract] OR electrocardiography[Title/Abstract] OR echocardiography[Title/Abstract] OR “cardiac magnetic resonance”[Title/Abstract] OR “computed tomography”[Title/Abstract] OR radiomics[Title/Abstract] OR haemodynamics[Title/Abstract] OR hemodynamics[Title/Abstract] OR biomarker[Title/Abstract] OR genomics[Title/Abstract] OR transcriptomics[Title/Abstract] OR proteomics[Title/Abstract] OR metabolomics[Title/Abstract] OR “multi-omics”[Title/Abstract] OR “non-coding RNA”[Title/Abstract] OR “single-cell”[Title/Abstract] OR “spatial omics”[Title/Abstract] OR “risk stratification”[Title/Abstract] OR “treatment response”[Title/Abstract] OR “precision medicine”[Title/Abstract] OR “digital twin”[Title/Abstract])).

English-language original investigations, systematic reviews, meta-analyses, consensus documents, and major guidelines addressing clinical detection, imaging or haemodynamic phenotyping, AI methodology, molecular mechanisms, biomarkers, omics, prognosis, or therapeutic stratification in pulmonary hypertension were considered. Because the aim was a broad narrative synthesis rather than exhaustive effect estimation, study selection was purposive. Priority was given to contemporary guidelines, landmark mechanistic studies, larger or multicentre cohorts, externally or prospectively validated AI models, studies with transparent methods and clinically relevant outcomes, and integrative molecular or multi-omics investigations supported by replication or biological validation. Earlier seminal publications were retained when required to establish key concepts. Conference abstracts, non-peer-reviewed reports, small case series, and studies with insufficient methodological detail were generally not prioritised. Because the included evidence spans diagnostic, prognostic, molecular, and interventional designs, no single formal risk-of-bias instrument was applied across all studies and no quantitative meta-analysis was performed. Instead, methodological quality was appraised qualitatively according to cohort representativeness, sample size and event burden, transparency of data handling and model development, internal and external validation, calibration where reported, biological replication or orthogonal validation for omics studies, and the clinical relevance of the outcome. These criteria informed the weight given to individual reports rather than serving as formal exclusion thresholds. Because study selection was purposive rather than exhaustive, this narrative review remains susceptible to selective citation; the qualitative appraisal should therefore not be interpreted as a formal study-by-study risk-of-bias or applicability assessment.

## 2. Artificial Intelligence in Pulmonary Hypertension: Concepts, Models, and Data Sources

AI methods in PH are useful chiefly because they can model non-linear relationships within and across heterogeneous data domains [18,19]. Supervised learning supports classification, haemodynamic estimation, and outcome prediction, whereas unsupervised learning can identify latent clinical or molecular subgroups [4,20]. Deep learning is suited for raw ECG and imaging data, while penalised regression and tree-based ensembles remain useful for structured datasets [21,22,23]. Model complexity must be matched to sample size and the intended task to limit overfitting in small, selected PH cohorts.

For precision PH, the key methodological issue is often integration rather than algorithm choice. Early integration combines harmonised features before model fitting; intermediate integration learns shared latent representations or cross-domain networks, and late integration combines modality-specific models or predictions. Multi-view approaches such as similarity-network fusion, multi-omics factor analysis, graph-based learning, and variational autoencoders are especially relevant when complementary information is distributed across molecular layers or across molecular, imaging, haemodynamic, and clinical domains. These architectures may capture cross-domain structure missed by single-modality models but increase vulnerability to missingness, scale imbalance, batch effects, and overfitting. Prediction is not causal inference: high predictive importance or explainable-AI attribution does not establish mechanistic involvement. Causal claims require an appropriate causal design and assumptions and, where feasible, biological or experimental validation.

Unsupervised clustering and latent-variable methods group patients according to similarity across clinical, haemodynamic, imaging, or molecular variables. For terminological consistency, phenotype denotes observable clinical, haemodynamic, imaging, or physiological characteristics, irrespective of whether their mechanism is known; endotype is reserved for a subgroup defined by a biologically meaningful or mechanistically supported disease process. A data-derived cluster should therefore be termed a phenotype or subgroup initially and considered a candidate endotype only after independent replication, temporal stability, mechanistic coherence across molecular and functional data, and clinically relevant prognostic or treatment-response associations [17,20,24].

The clinical validity of any AI model depends on the depth, scale, representativeness, and quality of its underlying data. Relevant sources include structured and unstructured electronic health records, ECG, echocardiography, CMR, computed tomography (CT), including CT pulmonary angiography, right-heart catheterisation waveforms and derived haemodynamics, laboratory biomarkers, multi-omics profiles, and wearable or remote-monitoring signals. Each modality provides a different view of the disease: electronic records capture longitudinal healthcare trajectories; ECG offers low-cost scalable screening; imaging characterises RV adaptation and pulmonary vascular remodelling; catheterisation provides the invasive physiological reference standard, and omics data resolve molecular heterogeneity [14,15,17]. The principal data modalities, computational tasks, and potential clinical outputs are summarised in Table 1. Therefore, throughout this review, we use multimodal integration for joint modelling across heterogeneous clinical, electrical, imaging, haemodynamic, biomarker, or molecular domains, whereas multi-omics integration is reserved for explicit joint analysis of at least two molecular omics layers. Incorporating an omics layer into a clinical or imaging model therefore makes the framework multimodal, but not necessarily multi-omics.

Within this framework, each modality occupies a different level of biological resolution rather than representing an isolated AI task. Genetic and transcriptomic data reflect susceptibility and active molecular programmes; proteomic and metabolomic signals capture downstream pathway activity; circulating biomarkers and haemodynamics quantify systemic and cardiopulmonary consequences, and ECG and imaging register organ-level expression in the pulmonary vasculature and right ventricle [14,17,24]. AI-based integration can link these layers, but assignment of a biological endotype requires concordance across modalities and should not be inferred from a single high-performing predictor.

## 3. AI for Early Detection, Screening, and Diagnostic Referral

The clinical question addressed in this section is: can AI identify individuals who should undergo further evaluation for PH? Early signals are often subtle, dispersed across routine investigations, and attributed to more common causes of breathlessness. AI may help integrate these otherwise overlooked signals and prompt earlier, with targeted investigation. Accordingly, ECG, echocardiography, chest imaging, electronic health records, and other modalities are considered here only insofar as they support case finding, triage, or referral; detailed phenotypic characterisation is addressed in Section 4.

These detectable signals can be understood as downstream manifestations of molecular and cellular injury: altered vascular-cell proliferation, inflammation, metabolic remodelling, and extracellular-matrix change increase pulmonary vascular load, whereas ECG and imaging record the resulting right-ventricular adaptation [3,4,14,17]. Early-detection algorithms therefore identify phenotypic consequences of disease biology rather than the molecular lesion itself. Their future value will depend on linking low-cost screening signatures to biomarker and omics data in deeply phenotyped cohorts, thereby testing whether an electrical or imaging signal enriches a reproducible biological endotype and a distinct therapeutic opportunity.

The standard 12-lead electrocardiogram is an attractive entry point because it is inexpensive, widely available, and often recorded repeatedly before PH is suspected. Deep-learning models, including convolutional neural networks, can analyse raw voltage–time waveforms and detect patterns beyond conventional signs of right-ventricular hypertrophy or strain. The PH Early-Detection Algorithm (PH-EDA), developed from Mayo Clinic ECG data and externally validated at Vanderbilt University Medical Centre, achieved areas under the receiver operating characteristic curve (AUCs) of 0.92 and 0.88, respectively. Its performance persisted for up to five years before diagnosis, with AUCs remaining at or above 0.79 and 0.73, respectively [25]. A separate University of California, San Francisco model achieved an AUC of 0.89 for overall PH and also identified pre-capillary PH, PAH, and group 3 PH, with AUCs of 0.91, 0.94, and 0.90, respectively. Applied to earlier ECGs, it retained an AUC of at least 0.79 up to two years before diagnosis [26]. In an earlier two-centre cohort, an AI-ECG algorithm achieved internal and external validation AUCs of 0.859 and 0.902. Among 2939 individuals without PH on baseline echocardiography, those classified as high-risk developed echocardiographic PH more often than low-risk individuals (31.5% versus 5.9%; *p* < 0.001) [27]. Importantly, the PH-EDA AUCs of 0.92 and 0.88 refer to discrimination of the study’s composite PH-likely reference label, defined by right-heart catheterisation (RHC) or echocardiographic criteria, in the Mayo test cohort and the external Vanderbilt cohort, respectively; they are not estimates restricted to RHC-confirmed PH. Likewise, the 31.5% versus 5.9% comparison was a secondary analysis of subsequent echocardiographic PH among the 2939 individuals without PH on baseline echocardiography and did not evaluate diagnostic delay, incident RHC-confirmed PH, or clinical utility.

Echocardiography remains the principal bridge between initial suspicion and invasive haemodynamic confirmation, although its performance depends on image quality, reader expertise, and a measurable tricuspid regurgitation velocity (TRV). In 7853 patients who underwent echocardiography and right-heart catheterisation within seven days, a gradient-boosting model integrating 19 echocardiographic features detected PH with an AUC of 0.83 and 88% sensitivity without requiring TRV [28]. In 1031 patients referred for suspected PH, automated TRV assessment matched manual diagnostic discrimination (AUC, 0.88) and yielded a value in 87% of studies [29]. However, automated TRV showed only modest discrimination in patients with milder haemodynamic abnormalities and did not outperform clinical interpretation [30]. These applications are relevant here because they refine the decision to proceed to expert evaluation or right-heart catheterisation; automated chamber segmentation and detailed right-ventricular phenotyping are discussed in Section 4.

The clinical value of AI may depend on pre-test probability. In systemic sclerosis, a random-forest model integrating pulmonary function, ECG, echocardiographic, and CT variables identified catheterisation-confirmed PH with an AUC of 0.92, 95% sensitivity, and 80% specificity; pulmonary artery diameter and diffusing capacity were the strongest predictors [31]. At the population level, a model based on five years of National Health Service resource-use data compared 709 patients with idiopathic PAH with more than 2.8 million controls and identified a disease-enriched subgroup, although sensitivity was only 14.1% at the selected rare-disease screening threshold [15]. These strategies are complementary: intensive multimodal assessment in high-risk populations and broad, low-cost case finding across electronic health records.

Other modalities can contribute to triage when their output changes the decision to pursue expert PH evaluation. In referred populations, a random-forest model distinguished PH due to left-heart disease from PAH, whereas CT algorithms identified parenchymal or chronic thromboembolic patterns that may redirect the diagnostic pathway [32,33,34]. A chest-radiograph deep-learning study evaluated PH detection across distinct validation datasets: in an RHC-confirmed internal cohort of 1158 patients, the CXR-PH-Net model achieved an AUC of 0.872 with sensitivity of 0.902, whereas in an external RHC-confirmed cohort of 90 patients from two hospitals the AUC was 0.811 with sensitivity of 0.803 [35]. These values therefore describe RHC-confirmed PH detection in separate internal and external cohorts and should not be compared directly with models using echocardiographic surrogate labels. The digital-stethoscope model addressed a different target, echocardiographically estimated pulmonary artery systolic pressure (PASP) ≥ 40 mmHg rather than RHC-confirmed PH: mean five-fold cross-validation AUC was 0.79 (range, 0.76–0.85), while the held-out test dataset yielded a per-recording AUC of 0.797 (95% CI, 0.75–0.84), sensitivity of 0.71, and specificity of 0.73 [36]. These studies are considered here as referral tools; neither performance estimate establishes shorter diagnostic delay nor clinical utility, and detailed characterisation of cardiac, pulmonary vascular, and lung phenotypes is reserved for Section 4.

The endpoint of this section is therefore not an algorithm-generated diagnosis or a complete phenotype, but an earlier, better-calibrated decision to escalate: from an abnormal AI-ECG, electronic-record, or low-cost imaging signal to structured echocardiography, and from intermediate or high echocardiographic probability to expedited expert-centre evaluation and right-heart catheterisation. Once suspicion or haemodynamic confirmation has been established, the task changes from detection to characterisation, which is the focus of Section 4. Current studies do not show that deployment shortens diagnostic delay or improves outcomes. Algorithms must therefore be prospectively tested in their intended populations, calibrated to local prevalence, and evaluated for false reassurance, referral burden, equity, clinical utility, and net benefit. Because these studies use different target conditions, reference standards, prediction horizons, and disease prevalences, their discrimination and threshold metrics are not directly comparable.

## 4. AI for Diagnostic Support and Cardiopulmonary Phenotyping

After screening or referral, the intended use changes from case finding to diagnostic support and quantitative characterisation. This section therefore asks whether AI can define the cardiovascular, pulmonary vascular, and lung phenotype in patients with suspected or confirmed PH. Structural adaptation of the right ventricle (RV) to increased pulmonary vascular load strongly influences symptoms, functional capacity, and survival. AI, machine learning (ML), and deep learning (DL) can extend imaging beyond visual assessment by standardising measurements, integrating complementary features, and identifying reproducible organ-level phenotypes.

To avoid overstating heterogeneous evidence, quantitative performance is reported with its study population, reference standard, validation setting, and comparator whenever these are available. Where the primary evidence is descriptive or does not provide a directly comparable same-cohort estimate, associations are described neutrally rather than ranked using qualitative terms such as ‘excellent’, ‘high’, or ‘strong’.

Conceptually, right-heart and pulmonary vascular imaging provides an intermediate phenotype between molecular pathology and clinical outcome. Endothelial dysfunction, dysregulated growth-factor signalling, inflammation, and metabolic remodelling are not directly visualised, but their cumulative consequences may be expressed as vascular pruning, altered flow, right-ventricular hypertrophy and dilation, impaired deformation, and right-ventricular–pulmonary-arterial uncoupling [3,4,14,17]. AI can quantify these organ-level patterns at scale and integrate them with haemodynamic and molecular data; imaging alone, however, cannot establish the causal molecular endotype.

### 4.1. Echocardiography

Within this characterisation phase, echocardiography provides detailed assessment of pulmonary pressure surrogates, RV morphology and function, and the cardiac consequences of elevated pulmonary vascular resistance. Rather than revisiting its role as a referral test, the focus here is automated quantification of RV dimensions, tricuspid annular plane systolic excursion (TAPSE), fractional area change (FAC), RV free-wall longitudinal strain, and indices of RV–pulmonary artery (RV–PA) coupling, which have diagnostic and prognostic significance in PH [37,38,39].

Despite its widespread availability, echocardiography is limited by operator dependence, variability in image quality, and imperfect correlation with invasive haemodynamic measurements obtained during right-heart catheterisation [14]. AI-based image analysis has the potential to address some of these limitations by improving reproducibility and enabling automated extraction of quantitative parameters.

One of the earliest applications of AI in PH imaging has been the automated segmentation of right-heart structures. Cervantes-Guzman et al. developed a deep learning segmentation framework based on the U-Net architecture that improved delineation of the right-sided chambers and demonstrated agreement with expert annotations [22]. Such approaches may facilitate standardised assessment of RV remodelling while reducing interobserver variability.

In patients already undergoing diagnostic evaluation, machine-learning analysis of echocardiographic and clinical features has been evaluated against RHC-based haemodynamic categories. Liao et al. studied 346 patients and reported an AUC of 0.945 in development and 0.950 (95% CI, 0.897–1.000) in an external cohort of 71 patients; the traditional echocardiographic assessment comparator had an AUC of 0.892 in the development cohort [40]. Hirata et al. analysed 885 patients and reported derivation AUCs of 0.789, 0.766, and 0.742 for non-PH, pre-capillary PH, and post-capillary PH, respectively; macro-classification accuracy was 59.4% versus 51.6% for guideline-based assessment in the derivation cohort, with prospective validation in 165 patients [41]. These same-cohort comparisons quantify discrimination or classification performance, but they do not by themselves establish incremental clinical utility or net benefit.

Among echocardiographic biomarkers, RV free-wall longitudinal strain (RVFWLS) has been studied as a marker of RV myocardial deformation and dysfunction. Across heterogeneous PH cohorts, impaired RV strain has been associated with invasive haemodynamic severity, exercise limitation, clinical worsening, and mortality [42,43,44,45]. Because these studies differ in population, acquisition method, software, and endpoint, the present review does not rank RV strain against TAPSE or FAC on the basis of qualitative descriptors alone. AI-enhanced speckle-tracking may support more automated and reproducible measurement, but incremental clinical value requires direct same-cohort comparison and external or prospective validation.

Despite these promising developments, several challenges remain. Variability in image acquisition, vendor-specific strain software, and the absence of universally accepted cut-off values continue to limit the standardisation of AI-enhanced echocardiographic biomarkers. Furthermore, most available studies have been conducted in relatively small cohorts, highlighting the need for larger prospective validation studies before widespread clinical implementation.

Overall, AI-based echocardiographic phenotyping may support more standardised quantification of RV adaptation and dysfunction. Whether these approaches provide clinically meaningful incremental diagnostic or prognostic value beyond conventional echocardiographic assessment requires same-cohort comparison, calibration, external validation, and prospective evaluation.

### 4.2. Cardiac Magnetic Resonance

Cardiac magnetic resonance (CMR) is the reference standard for non-invasive assessment of RV structure and function. It provides highly reproducible measurements of RV volumes, ejection fraction, ventricular mass, and interventricular interactions while also allowing evaluation of pulmonary arterial remodelling [46]. CMR-derived RV parameters have established associations with outcome in PH [47,48,49].

The application of AI to CMR has primarily focused on automated image analysis and extraction of quantitative imaging biomarkers. By integrating anatomical and physiological parameters, AI-enhanced CMR has been evaluated for identifying PH and characterising disease severity. In one representative study, MRI-derived structural features combined with computational physiological metrics provided diagnostic information within the study cohort [50]. In the absence of a consistently reported same-cohort comparator and external validation across such models, these findings are interpreted as evidence of feasibility rather than proof of incremental clinical utility.

Radiomics and ML techniques have further expanded the information obtainable from CMR. Quantitative features extracted from the RV and left ventricular myocardium have identified imaging patterns associated with PH, including in patients with relatively preserved ventricular function [51]. These findings support investigation of imaging biomarkers not captured by conventional measurements, but the magnitude and transportability of diagnostic performance should be judged from study-specific estimates rather than qualitative descriptors.

CMR-based ML has also been evaluated for prognosis and haemodynamic phenotyping. In 256 newly diagnosed patients with PH, three-dimensional RV motion analysis improved one-year survival discrimination compared with conventional markers in the same study (AUC of 0.73 versus 0.60) [16]. Automated CMR measurements have also shown associations with invasive haemodynamic measurements and mortality [23]. These results arise from different tasks and datasets and therefore support quantitative phenotyping rather than a single general claim of diagnostic or prognostic superiority.

Parameters such as RV ejection fraction, RV end-diastolic volume, ventricular mass index, right atrial enlargement, and interventricular septal displacement consistently emerge as important markers of disease severity and prognosis [47,48,49]. Consequently, AI-enhanced CMR may facilitate more comprehensive and reproducible characterisation of RV adaptation and disease progression.

Nevertheless, important barriers remain. Most AI-based CMR models have been developed and validated in specialised referral centres, and their performance in broader real-world populations remains uncertain. In addition, differences in acquisition protocols, scanner vendors, and segmentation methodologies may affect model generalisability. Future studies should prioritise external validation and assessment of incremental value over established CMR risk markers.

### 4.3. Computed Tomography and Radiomics

Here, computed tomography (CT) is considered primarily as a phenotyping and treatment-planning platform rather than as a screening test. It provides detailed assessment of pulmonary vascular anatomy, cardiac remodelling, associated lung disease, and chronic thromboembolic obstruction [33,34,46,52]. It is therefore particularly valuable for defining the pulmonary and vascular context in which PH occurs and remains central to diagnosis and therapeutic planning in chronic thromboembolic pulmonary hypertension (CTEPH) [52].

Traditional CT interpretation relies largely on visual assessment and a limited number of anatomical measurements, such as pulmonary artery diameter and the pulmonary artery-to-aorta ratio. Recent advances in quantitative CT and radiomics have enabled the automated extraction of high-dimensional imaging features that describe pulmonary vascular morphology, cardiac remodelling, and lung parenchymal abnormalities [53].

Machine-learning algorithms can quantify vascular calibre, branching patterns, vessel tortuosity, and vascular pruning, generating objective descriptors of pulmonary vascular morphology that have been associated with invasive haemodynamic measures in the studied cohorts [54,55,56]. Similarly, radiomic analysis of lung parenchyma may support characterisation of coexisting pulmonary disorders and CT-derived phenotyping in pulmonary vascular disease [57,58].

In patients with CTEPH, AI-assisted CT analysis has been investigated for automated detection of chronic thromboembolic lesions and quantitative assessment of vascular obstruction. Dual-energy CT and iodine perfusion mapping can additionally characterise pulmonary vascular anatomy and regional perfusion, but the incremental diagnostic or treatment-planning value of AI-enabled analyses requires study-specific validation against appropriate reference standards.

However, radiomics studies remain particularly vulnerable to variability in image acquisition, reconstruction algorithms, and feature extraction methods. The lack of standardised radiomic pipelines currently limits reproducibility across institutions and represents a major obstacle to clinical translation.

### 4.4. Integration of Imaging, Haemodynamic, and Clinical Data

The greatest potential of AI-based imaging phenotyping lies in integrating imaging features with invasive haemodynamics, biomarkers, and clinical variables. Contemporary ML models increasingly combine imaging-derived parameters with mean pulmonary artery pressure (mPAP), pulmonary vascular resistance (PVR), right atrial pressure (RAP), cardiac index, mixed venous oxygen saturation, functional status, and laboratory biomarkers.

Evidence for multimodal models in PH remains limited and task-specific. In the study by Dawes et al., 256 patients with newly diagnosed PH underwent CMR, right-heart catheterisation, and functional assessment; adding machine-learned three-dimensional RV motion to conventional imaging, haemodynamic, functional, and clinical markers improved one-year survival discrimination within the same cohort under eight-fold cross-validation (AUC of 0.73 versus 0.60; *p* < 0.001) [16]. By contrast, the quantitative CT study by Rahaghi et al. compared pulmonary vascular morphology in 18 patients with CTEPH and 15 dyspnoea controls and explored associations with invasive haemodynamics; it did not evaluate a validated multimodal model against a conventional prognostic score [54]. These studies therefore support quantitative phenotyping and, in the CMR study, incremental prognostic discrimination, but they do not justify a general claim that integrated models outperform conventional risk assessment.

This integrative step is a conceptual bridge in the proposed molecular-to-therapeutic sequence. A candidate endotype should be supported by convergence between a biological signal, such as an immune, growth-factor, or metabolic programme; a measurable organ phenotype, such as vascular pruning or maladaptive right-ventricular remodelling; and a reproducible clinical trajectory [17,24]. Cross-modal concordance can strengthen biological plausibility and generate testable treatment hypotheses, but neither multimodal prognostic improvement nor imaging–haemodynamic association establishes a mechanistic endotype or predicts treatment benefit.

Although the results are encouraging, most available studies remain retrospective, frequently involve limited sample sizes, and often lack independent external validation. Demonstrating that AI-driven phenotyping improves clinical decision-making and patient outcomes beyond current guideline-based approaches remains a critical unmet need. Future research should therefore focus on prospective multicentre evaluation, model interpretability, calibration, and assessment of real-world clinical utility before routine implementation can be recommended.

Section 4 therefore addresses characterisation after suspicion or confirmation: AI-derived imaging features define observable cardiovascular and pulmonary phenotypes that may support diagnosis and prognosis. They do not by themselves establish a biological endotype or a treatment-selection rule. The next step, considered in Section 5, is to integrate these organ-level phenotypes with molecular and multi-omics data to test for mechanistically supported endotypes.

The distinction between an imaging phenotype and a molecular endotype is therefore a distinction between how and why PH is expressed. Imaging phenotyping describes the anatomical and functional manifestations of disease—for example, pulmonary vascular pruning and remodelling, RV hypertrophy or dilatation, impaired myocardial deformation and RV–pulmonary-arterial uncoupling, fibrotic tissue signatures, and perfusion heterogeneity—whereas molecular endotyping asks which biological programmes generate those manifestations. Cross-modal integration can test whether such imaging features serve as non-invasive surrogate phenotypes of endothelial proliferative signalling, inflammation, extracellular-matrix turnover, or altered cardiopulmonary metabolism [14,17,24]. These relationships must be demonstrated rather than assumed: an imaging signature should be regarded as a mechanistic surrogate only when it is reproducibly associated with a defined molecular pathway across independent cohorts and ideally predicts differential response to pathway-directed therapy.

## 5. AI, Multi-Omics, and Molecular Endotyping in Pulmonary Hypertension

Section 4 described how PH is expressed anatomically and functionally. This section asks why those phenotypes develop by examining the molecular programmes that shape pulmonary vascular remodelling and RV adaptation. It therefore provides the biological layer of the framework introduced above. Its purpose is not to treat molecular biology as a separate domain, but to define the mechanisms where AI models connect to electrical, imaging, haemodynamic, biomarker, prognostic, and treatment-response phenotypes [17,24]. The central translational question is whether a data-derived subgroup represents a reproducible mechanism that can be measured in practice and linked to differential outcome or therapeutic response.

### 5.1. Molecular Heterogeneity as the Biological Basis for Endotyping

Pulmonary hypertension (PH) is defined haemodynamically, but its biological substrate is markedly heterogeneous. This distinction is essential for precision medicine: patients who share similar pulmonary pressures and functional limitation may have different initiating mechanisms, vascular cell states, inflammatory profiles, metabolic adaptations, and patterns of right-ventricular (RV) response. Most molecular and multi-omics evidence currently derives from pulmonary arterial hypertension (PAH), particularly idiopathic and heritable PAH; therefore, extrapolation to PH associated with left-heart disease, lung disease, or chronic thromboembolic obstruction should remain cautious. Within PAH itself, the conventional clinical classification captures aetiology but only partially reflects the molecular processes that determine disease penetrance, progression, and treatment response.

Genomic studies established impaired bone morphogenetic protein receptor type 2 (BMPR2) signalling as a central susceptibility mechanism in heritable PAH [59]. Subsequent large-scale sequencing expanded the genetic architecture to include rare variants in genes involved in transforming growth factor-β (TGF-β) signalling, endothelial integrity, ion-channel function, transcriptional regulation, and pulmonary vascular development, including ACVRL1, ENG, SMAD9, GDF2, SOX17, TBX4, KDR, ATP13A3, and AQP1 [60]. Genome-wide association analyses further identified common variation near SOX17 and within the HLA region, linking pulmonary vascular development and immune regulation to both disease susceptibility and outcome [61]. However, incomplete penetrance among mutation carriers demonstrates that genotype alone is insufficient to define the disease state. Sex, age, hormonal exposure, inflammation, environmental stressors, somatic events, and epigenetic regulation probably interact with inherited susceptibility to determine whether and when clinically manifest PAH develops.

At pathway level, PAH is characterised by an imbalance between growth-restrictive BMP-SMAD1/5/9 signalling and proliferative TGF-β/activin-SMAD2/3 signalling. Experimental work has shown that endothelial activin A can promote BMPR2 internalisation and degradation, thereby amplifying endothelial dysfunction [62]. Conversely, ligand trapping with ACTRIIA-Fc rebalanced activin/growth differentiation factor signalling against BMP signalling, reduced vascular-cell proliferation, and reversed pulmonary vascular remodelling in experimental models [63]. These findings illustrate how molecular endotyping may identify disease-driving axes that are not evident from haemodynamics alone. Nevertheless, the BMPR2–activin axis is only one component of a broader network that includes reduced nitric oxide and prostacyclin signalling, enhanced endothelin activity, mitochondrial dysfunction, oxidative stress, dysregulated iron handling, extracellular-matrix remodelling, and immune-cell activation.

Non-coding RNAs add a further regulatory layer. Reduced miR-204 expression in pulmonary artery smooth muscle cells promotes a proliferative and apoptosis-resistant phenotype through STAT3-NFAT signalling [64]. Network-based analyses identified miR-21 as an integrator of multiple pathogenic pathways, including hypoxia and BMPR2-related signalling [65], whereas loss of endothelial miR-124 shifts pyruvate kinase splicing towards PKM2 and supports glycolytic, proliferative metabolism [66]. These observations support a model in which molecular heterogeneity arises not from a single dominant lesion, but from interacting genetic, epigenetic, transcriptional, inflammatory, and metabolic programmes. An endotype should therefore denote a reproducible, biologically coherent mechanism with potential prognostic or therapeutic relevance, rather than a statistically convenient cluster without mechanistic validation.

### 5.2. Omics Layers and Levels of Integration

Each omics layer interrogates a different level of PAH biology. Genomics identifies inherited or acquired susceptibility but is relatively static. Bulk transcriptomics measures active gene-expression programmes, although signals may be confounded by differences in circulating-cell composition or tissue sampling. Proteomics is closer to functional biology and can capture secreted mediators, extracellular-matrix turnover, inflammation, and cardiac stress. Metabolomics and lipidomics provide a dynamic readout of cellular bioenergetics and systemic adaptation. MicroRNA and other non-coding RNA profiles reflect post-transcriptional regulation, while single-cell and spatial technologies resolve cell-specific states and cellular interactions that are obscured in bulk tissue.

For terminological precision, profiling several omics modalities in parallel is not synonymous with multi-omics integration. A study uses multiple omics approaches when each layer is analysed separately and the results are compared narratively. True multi-omics integration requires an explicit joint analytical step across at least two molecular layers such that the resulting biological inference depends on their combined information. As outlined in Section 2, integration may occur at early, intermediate, or late stages. Linking omics outputs to imaging or haemodynamics extends the framework to multimodal, rather than strictly multi-omics, integration.

#### 5.2.1. Single-Omics and Cell-Resolved Evidence

Circulating proteomics has produced some of the most clinically advanced examples. Aptamer-based profiling identified a nine-protein panel associated with mortality independent of conventional risk markers [67]. A later analysis using a broader proteomic platform derived and replicated a six-protein score that improved long-term risk discrimination beyond N-terminal pro-B-type natriuretic peptide and appeared sensitive to treatment-related change [68]. These studies demonstrate the value of high-dimensional molecular data, but they also highlight an important distinction: a prognostic biomarker panel may improve risk prediction without necessarily defining a causal endotype. Proteins associated with adverse outcome may reflect pulmonary vascular remodelling, RV dysfunction, renal impairment, systemic inflammation, or treatment intensity. Their mechanistic interpretation therefore requires integration with tissue expression, cell-of-origin data, and experimental validation.

Metabolomic studies consistently indicate altered bioenergetics in PAH. Plasma profiling has linked disease outcome to modified transfer-RNA nucleosides, altered glycolysis, fatty-acid oxidation, and mitochondrial pathways [69]. More recent analyses identified metabolite signatures associated with RV dilation, disease severity, and mortality, including changes in lipid, amino-acid, and nucleotide metabolism [70]. Metabolomics is particularly attractive for longitudinal monitoring because it responds rapidly to physiological and therapeutic perturbations. However, it is also vulnerable to diet, fasting status, renal and hepatic functions, microbiome composition, concomitant medication, and sample-processing conditions. Robust clinical translation therefore requires standardised pre-analytical procedures and external validation across geographically and phenotypically diverse cohorts.

Single-cell transcriptomics and spatially resolved profiling have substantially refined the cellular map of PAH. Human lung studies have identified disease-associated transcriptional programmes in endothelial cells, pericytes, smooth muscle cells, fibroblasts, and macrophages, including pathways related to extracellular-matrix organisation, angiogenesis, inflammation, and cell survival [71]. Single-cell analysis of pulmonary artery endothelial cells has further revealed endothelial subpopulations and patient-specific activation states [72]. Cross-species computational integration of single-cell datasets from experimental models with human PAH has been used to prioritise conserved pathways and candidate drugs [73]. Spatial single-cell imaging has shown that inflammatory subsets are not randomly distributed but are anatomically linked to vascular lesions, with monocyte-derived dendritic cells, neutrophils, and specific T-cell populations associated with more severe vasculopathy [74]. These technologies move the field from average tissue expression towards cell-state and cell-neighbourhood biology, which is more compatible with mechanistic endotyping.

The RV should be considered an additional molecular compartment rather than a passive consequence of pulmonary vascular disease. RV adaptation varies substantially among patients exposed to comparable afterload. Human tissue, imaging, and metabolic studies have shown suppressed fatty-acid oxidation, myocardial lipid accumulation, and lipotoxic intermediates in heritable and non-heritable PAH [75,76]. High-dimensional plasma proteomics has also identified extracellular-matrix, inflammatory, and metabolic signatures associated with RV dilation, natriuretic peptide levels, and mortality, with supporting evidence from RV tissue datasets [77]. An integrated molecular model of PH should therefore distinguish observable pulmonary vascular and RV-adaptation phenotypes and then determine whether each is explained by distinct, mechanistically supported endotypes, while recognising that the two compartments interact through haemodynamic load, neurohormonal activation, inflammation, and systemic metabolism.

Table 2 provides a study-level evidence map of representative molecular and omics investigations, explicitly distinguishing human PAH cohorts, experimental PH models, lung and RV tissue, isolated vascular cells, and circulating biospecimens. Table 3 separately identifies the limited set of completed integrative PH studies and specifies the level at which multi-omics or molecular–imaging integration was performed. This distinction is important: evidence from single-omics discovery, cross-species mechanistic studies, completed integrative analyses, and the proposed future architecture should not be conflated. Figure 2 depicts the latter target architecture.

#### 5.2.2. Completed Integrative Studies

Human PH evidence meeting this stricter definition remains limited. Hong et al. integrated multicentre PAH lung transcriptomics with clinicopathological phenotyping, genome-wide association data, Bayesian regulatory networks, single-cell transcriptomics, and pharmacotranscriptomics to prioritise asporin as a protective network hub [78]. Khassafi et al. linked human and experimental RV transcriptomes to plasma proteomics and MRI-defined RV adaptation states, identifying extracellular-matrix proteins associated with maladaptive remodelling and outcome [79]. In high-altitude PH, serum proteomic and metabolomic data were integrated at pathway level, highlighting coordinated lipid, immune, cytoskeletal, and oxidative-stress alterations [80]. These examples span cross-cohort systems integration and within-participant joint profiling, but they remain discovery-oriented and do not yet establish externally validated molecular endotypes or treatment-response classifiers. Most PH reports still analyse one omics layer at a time or compare layers post hoc; therefore, the evidence supports describing the field as emerging multi-omics approaches, with true integration as a developing subset rather than a mature clinical capability.

#### 5.2.3. Proposed Future Multi-Omics and Multimodal Architecture

The architecture proposed in this review begins where the completed studies above end. It assumes prospective, preferably within-participant acquisition of complementary omics layers, an explicit joint integration step, and subsequent linkage to imaging, haemodynamic, clinical, and treatment trajectories. Translation would then require independent replication, calibration, mechanistic validation, and prospective testing of clinical utility or treatment-by-signature interactions. No PH study currently satisfies this complete chain. Figure 2 should therefore be interpreted as a target translational architecture rather than as an established clinical workflow.

### 5.3. Artificial Intelligence for Molecular Phenotype Discovery and Candidate Endotyping

Artificial intelligence (AI) and machine-learning methods are useful in molecular phenotype discovery because omics datasets contain thousands of correlated variables, non-linear interactions, and often more features than patients. Supervised models can select molecular features that predict mortality, clinical worsening, or therapeutic response, but such predictive associations do not establish causal involvement in PH pathobiology. Unsupervised clustering and network-based methods can identify previously unrecognised patient groups without imposing conventional clinical labels. The integrative approaches introduced in Section 2 can then combine complementary molecular layers; joint modelling of at least two molecular omics layers constitutes multi-omics integration, whereas extension to imaging, haemodynamic, or clinical domains is multimodal.

Two single-omics clustering studies illustrate molecular heterogeneity in PAH but should be interpreted as cluster-discovery studies rather than as validated endotyping. Sweatt et al. applied unsupervised consensus clustering to circulating immune mediators and identified four cytokine-defined PAH clusters associated with different inflammatory profiles, clinical characteristics, and transplant-free survival [17]. The robustness of the cross-sectional cluster solution was assessed using the resampling and consensus procedures reported in the study, while demographic characteristics, PAH subtype, treatment exposure, and markers of disease severity were examined across the resulting groups. These analyses do not establish that cluster assignment is independent of age, sex, treatment, disease severity, or centre-related effects, and a circulating-protein panel does not directly resolve differences in blood-cell composition. Most importantly, longitudinal stability of individual cluster assignment and treatment-by-cluster interactions were not demonstrated. We therefore refer to these groups as immune-protein clusters or molecular phenotypes rather than established endotypes.

Kariotis et al. used unsupervised whole-blood transcriptomic profiling to identify three major idiopathic-PAH molecular clusters with distinct gene-expression programmes and clinical associations [24]. The transcriptomic workflow included cluster-stability and sensitivity analyses and explicitly examined technical or batch structure and estimated blood-cell composition; demographic, treatment, and disease-severity variables were also evaluated in relation to cluster membership. These safeguards strengthen the cross-sectional biological signal but do not eliminate residual confounding by clinical or centre-related factors, nor do they demonstrate prospective temporal stability or differential treatment response. Accordingly, these groups are described here as transcriptomic clusters or molecular phenotypes and at most candidate endotypes pending independent longitudinal, mechanistic, and treatment-response validation.

The following cross-layer examples are conceptual validation patterns rather than established PH endotypes. Multi-omics integration may move candidate molecular phenotypes towards better-supported endotypes by requiring concordance across biological levels. For example, a putative inflammatory endotype could be supported by a cytokine pattern, immune-cell transcriptomic state, spatial immune infiltration, and a corresponding clinical trajectory. A metabolic endotype could combine circulating metabolites, RV lipid imaging, tissue expression of fatty-acid oxidation genes, and functional measures of RV reserve. Such cross-layer consistency reduces the risk that a model captures platform-specific noise. It may also improve interpretability by organising thousands of features into pathway-level representations. Network approaches are particularly useful because they can identify central regulators or modules rather than isolated biomarkers, thereby supporting target prioritisation and drug-repurposing analyses.

Methodological rigour is critical. The combination of small cohorts and high-dimensional data creates a substantial risk of overfitting, unstable feature selection, and optimistic performance estimates. Feature selection, normalisation, imputation, and hyperparameter tuning must be performed within resampling loops to prevent information leakage. Cluster stability should be assessed across algorithms and subsamples, and models should undergo external validation in independent cohorts using harmonised assays. Analyses should explicitly address treatment exposure, centre effects, ancestry, comorbidities, and sample timing. Where possible, models should estimate uncertainty and provide interpretable pathway or feature contributions. Longitudinal sampling is also necessary because molecular states may evolve with disease progression or therapy. A temporally variable molecular pattern should be described as a state or phenotype unless it retains a coherent mechanistic basis; the activity of an endotype may change over time, but its defining biological mechanism should remain reproducible.

### 5.4. Biological Validation of Candidate Endotypes

Candidate molecular endotypes emerging from AI or multi-omics analysis require biological validation before any prognostic or therapeutic interpretation. Validation should establish reproducibility across independent cohorts, mechanistic coherence across molecular and functional data, and either temporal stability or biologically interpretable state transitions. Concordance across omics layers, tissues, and pulmonary vascular or right-ventricular phenotypes is particularly important because a statistically robust cluster may still reflect disease severity, treatment exposure, cell-composition differences, or technical batch effects rather than a distinct disease mechanism.

Biological plausibility should be strengthened through causal inference, experimental perturbation, and replication in relevant models. Tissue-based discovery should also be linked to scalable blood-based or imaging surrogates, because lung and right-ventricular tissue are rarely available during routine care. Mechanistically informed drug development, exemplified by activin/BMP pathway studies leading to ligand-trap efficacy [63,81], does not by itself establish a predictive endotype; designation requires evidence that the molecular state identifies differential treatment benefit.

A credible validation pathway should therefore proceed from AI-enabled discovery of molecular phenotypes to independent replication, cross-omics and cross-tissue confirmation, mechanistic testing, and development of parsimonious, clinically measurable biomarkers. Only after these steps should a candidate endotype be advanced to prognostic evaluation or to a treatment-response hypothesis. The therapeutic and clinical-trial implications of validated endotypes are considered in Section 7.

## 6. AI for Prognosis and Risk Stratification

Accurate risk stratification is central to PAH management because it informs therapeutic escalation, monitoring intensity, transplantation referral, and the interpretation of treatment response [5,82]. Validated tools such as REVEAL 2.0 and REVEAL Lite 2, COMPERA-based models, and the ESC/ERS framework have improved evidence-based prognostication. These tools should be evaluated against their intended prognostic use in PAH rather than against distinct tasks such as aetiological classification, mixed-group phenotyping, or molecular endotyping. Nevertheless, conventional point-based systems reduce complex trajectories to a limited set of variables, generally assume relatively simple functional relationships, and may be difficult to apply when data are missing or collected asynchronously [4,5,82].

ML methods can accommodate non-linear interactions, higher-dimensional inputs, and conditional dependencies among variables. The Pulmonary Hypertension Outcomes Risk Assessment (PHORA) model uses a tree-augmented naïve Bayesian network built from REVEAL 2.0 clinical, functional, and haemodynamic variables [83]. In the primary report, model development and internal evaluation used REVEAL data with 10-fold cross-validation, whereas transportability was assessed in two registries independent of model development: COMPERA (*n* = 3849) and the Pulmonary Hypertension Society of Australia and New Zealand registry (PHSANZ; *n* = 978). For one-year mortality/survival discrimination, PHORA yielded an AUC of 0.80 in REVEAL, compared with 0.76 for REVEAL 2.0 in the same internal analysis, and AUCs of 0.74 and 0.80 in COMPERA and PHSANZ, respectively [83]. The primary performance summary did not provide a uniform set of 95% confidence intervals, one-year event counts, formal calibration slope/intercept, or threshold-specific sensitivity, specificity, PPV, and NPV across all three cohorts; these quantities are therefore identified as not reported in Table 4 rather than inferred. Survival separation across prespecified risk categories was reported, but this should not be equated with a full calibration analysis. Thus, the external registries support transportability of discrimination, while incremental clinical utility over contemporary PAH risk tools remains to be demonstrated prospectively.

Advanced imaging provides prognostic information that is not fully represented by conventional risk scores. In 256 newly diagnosed patients with PH, supervised analysis of three-dimensional CMR motion identified adverse patterns involving reduced septal, free-wall, and basal longitudinal contraction. Integration of these motion signatures with clinical and haemodynamic variables improved survival discrimination compared with conventional markers alone [16]. Automated CMR measurements have also shown associations with invasive haemodynamics and mortality, supporting reproducible incorporation of RV structure and function into multimodal risk models [23].

Unsupervised ML adds a complementary dimension by identifying latent phenotypes with distinct trajectories. Consensus clustering of circulating immune proteins identified four cytokine-defined PAH clusters with different transplant-free survival [17]. Clustering based on routinely available variables has likewise identified groups with differing mortality profiles [20]. These findings are hypothesis-generating: cluster robustness within a cross-sectional dataset does not establish temporal stability, a defining biological mechanism, or treatment-effect modification. Age, sex, treatment exposure, disease severity, centre, blood-cell composition, and technical batch may contribute to apparent cluster structure and should be addressed explicitly in replication studies. Accordingly, these groups are treated as molecular or clinical phenotypes rather than endotypes unless a reproducible mechanism has been demonstrated.

Within the proposed framework, prognosis should be regarded as an emergent property of observable pulmonary vascular and right-ventricular adaptation phenotypes, which may reflect one or more underlying endotypes. AI models that combine molecular pathway activity with organ-level phenotypes may distinguish patients who share a conventional risk category but reach it through different biological trajectories [17,20,24,77]. However, these groups should remain classified as prognostic phenotypes unless a reproducible mechanism is demonstrated; clinical relevance additionally requires calibrated prognostic information and ultimately evidence of differential treatment response rather than merely another risk cluster. At present, however, molecularly anchored prognostic models are supported predominantly by PAH cohorts; their transportability to PH associated with left-heart disease, lung disease, or chronic thromboembolic obstruction remains unestablished.

Longitudinal prognostic models should integrate serial clinical status, imaging, biomarkers, exercise capacity, and invasive haemodynamics rather than rely on a single baseline snapshot. Prespecified prognostic outcomes may include mortality, hospitalisation, clinical worsening, and transplantation, with calibration, uncertainty, subgroup performance, and net clinical benefit reported alongside discrimination. Whether a prognostic model changes management safely and improves patient-centred outcomes are separate implementation questions addressed in Section 8.

Because discrimination, sensitivity, specificity, and predictive values are conditional on the target condition, reference standard, prediction horizon, and prevalence, performance estimates should be interpreted within the clinical task for which each model was developed. Table 4 therefore summarises selected key studies using the original study definitions and reports unavailable quantities as not reported rather than imputing or back-calculating them.

## 7. AI for Prediction of Treatment Benefit and Therapeutic Stratification in Pulmonary Arterial Hypertension

Pulmonary arterial hypertension (PAH) is characterised by substantial variability in progression and treatment response despite contemporary multiparametric risk assessment [5,82]. The evidence considered in this section is therefore PAH-specific unless stated otherwise, with molecular data concentrated especially in idiopathic and heritable PAH. Section 6 addressed prognosis irrespective of treatment; the distinct question here is whether a reproducible phenotype, biomarker signature, or biologically validated endotype can demonstrate differential benefit from a specified intervention. Therapeutic or molecular inferences from PAH are not extrapolated to PH groups 2–5 without disease-specific evidence.

### 7.1. Prediction of Treatment Benefit: Evidence Requirements and Research Agenda

A prognostic association, patient cluster, biomarker-outcome relationship, or higher AUC does not demonstrate prediction of benefit from a specific therapy. A treatment-response phenotype denotes a reproducible response pattern under a defined intervention, whereas a predictive endotype additionally requires a mechanistically supported subgroup with reproducible treatment-effect modification [17,24,63,81]. Accordingly, a model remains prognostic unless it shows that the effect of a prespecified treatment differs across the identified signature or subgroup.

The evidentiary hierarchy should therefore be explicit. Association or discrimination can generate a treatment-response hypothesis, but therapeutic prediction requires a properly evaluated treatment-by-biomarker, treatment-by-signature, or treatment-by-endotype interaction, preferably within randomised data. A candidate predictor should then undergo independent validation with calibration and clinically relevant performance assessment. Before implementation, a prospective decision-impact study must establish that model-guided management changes decisions appropriately and improves patient-centred outcomes without unacceptable safety consequences. Without this sequence, an AI model should not be used to select, withhold, escalate, or sequence PAH therapy.

Multimodal models can be designed to combine clinical variables, biomarkers, echocardiography, cardiopulmonary exercise testing, right-heart catheterisation, CMR, and molecular signatures [18,19,84]. Such integration may identify treatment-associated patterns, but unless development data contain an appropriate treatment contrast and the model demonstrates treatment-effect heterogeneity, the output cannot be interpreted as an estimate of individual therapeutic benefit.

The expanding PAH therapeutic landscape provides clinically important candidate settings in which predictive models could be tested. Endothelin-receptor antagonists, phosphodiesterase type-5 inhibitors, prostacyclin-pathway therapies, soluble guanylate cyclase stimulators, and activin signalling-targeted therapy have demonstrated efficacy in clinically defined PAH populations [5,81,85]. These population-level treatment effects do not establish that an AI-derived phenotype or molecular signature can select the drug or treatment sequence that will benefit an individual patient.

The activin/BMP axis illustrates this distinction. Genetic and experimental studies identified an imbalance between growth-restrictive BMP signalling and proliferative activin/TGF-β signalling, and ligand trapping subsequently demonstrated clinical efficacy with sotatercept in PAH [63,81,85]. This sequence validates molecularly informed therapeutic development, but it does not establish a biomarker-defined responder population. Current evidence therefore does not support selecting sotatercept on the basis of a molecular endotype. Future randomised studies should embed prospective biospecimen collection and test whether baseline or early-change signatures identify differential treatment benefit.

Upfront combination therapy, escalation to triple therapy or parenteral prostacyclins, sotatercept initiation, and referral for lung transplantation should therefore be framed as target decisions for future trials rather than as outputs of existing AI models [5,86]. For each candidate decision, the biomarker or model and its decision threshold should be prespecified before treatment allocation; the study should include a randomised comparison of clinically relevant treatment strategies when a therapeutic effect is being predicted, formally test a treatment-by-biomarker or treatment-by-model interaction, reproduce the interaction in an independent external cohort, and then evaluate whether model-guided management improves patient-relevant benefit without unacceptable harm. No such evidentiary sequence currently supports AI-guided selection, escalation, sequencing, or withholding of PAH therapy [5,18,19,87].

To extend the activin/BMP example across the major therapeutic axes, Table 5 applies a common translational template: molecular pathway → measurable biomarker or omics signature → candidate AI-defined endotype → therapeutic implication → current evidence status. The endotype labels are deliberately conditional; they denote hypotheses that require independent replication, mechanistic coherence, temporal stability, and prospective demonstration of treatment-effect modification. Because the pathway-directed therapeutic evidence summarised below derives predominantly from PAH, these molecular-to-therapy mappings should not be extrapolated to other PH groups without disease-specific validation.

Evidence status is described qualitatively rather than as a formal GRADE hierarchy. ‘Established clinical application’ indicates that a pathway-directed therapy has demonstrated efficacy in clinically defined PAH populations; it does not imply that a biomarker or AI model can select responders. ‘Emerging association’ denotes biologically coherent, preferably replicated molecular evidence without a validated treatment interaction. ‘Investigational hypothesis’ denotes discovery-stage evidence that remains insufficient for therapeutic stratification.

Across the first four pathways, clinical efficacy of the drug class is established, but predictive molecular stratification is not. Accordingly, the candidate AI-defined endotypes in Table 5 are testable hypotheses for prospective treatment-by-endotype interaction studies and trial enrichment, not recommendations for current prescribing.

### 7.2. AI-Enabled Response Monitoring and Clinical-Trial Enrichment

AI-enabled response monitoring and clinical-trial enrichment are plausible near-term extensions of therapeutic stratification. Wearable and remote-monitoring platforms can provide repeated physiological measurements that may detect early change after treatment, but PH-specific evidence remains limited and no AI-guided monitoring strategy has yet demonstrated improved clinical outcomes [18,19]. These tools should therefore be evaluated as adjuncts to established clinical, imaging, and haemodynamic follow-up rather than as autonomous triggers for treatment modification. In particular, an AI-derived deterioration signal should not trigger treatment escalation itself; the candidate therapeutic decisions and evidentiary requirements for future predictive trials are defined in Section 7.1.

Clinical-trial enrichment is a distinct near-term research application. Prognostic enrichment selects patients with a sufficiently high event rate, whereas predictive enrichment selects those with the target pathway or cellular state most likely to respond. Molecular signatures may also identify early non-responders, support adaptive randomisation, or serve as pharmacodynamic markers [18,86]. Enrichment algorithms should be evaluated across sex, ancestry, age, PAH subtype, and background therapy and should demonstrate incremental utility over simpler clinical models.

More speculative architectures, including multimodal foundation models and digital twins, are considered in Section 9 rather than within the current or near-term therapeutic evidence base.

### 7.3. Translational Perspective

The translational pathway can therefore be stated explicitly: biologically validated endotype or reproducible signature → prespecified treatment-response hypothesis → demonstrated treatment-effect interaction → independent validation → prospective decision-impact evaluation. AI may support discovery and measurement at each stage, but it does not collapse this evidentiary sequence. The current molecular-to-therapeutic framework remains a PAH-focused research agenda rather than a PH-wide or clinically implemented therapeutic paradigm [18,19,84,86,87,88].

To make this scope explicit, Table 6 maps each PH group to the principal clinical tasks addressed in the current literature, the predominant data modalities, and the qualitative maturity of the evidence. The map is intended to prevent evidence generated in PAH—particularly idiopathic and heritable PAH—from being interpreted as directly transferable to other PH groups.

**Figure 3:**
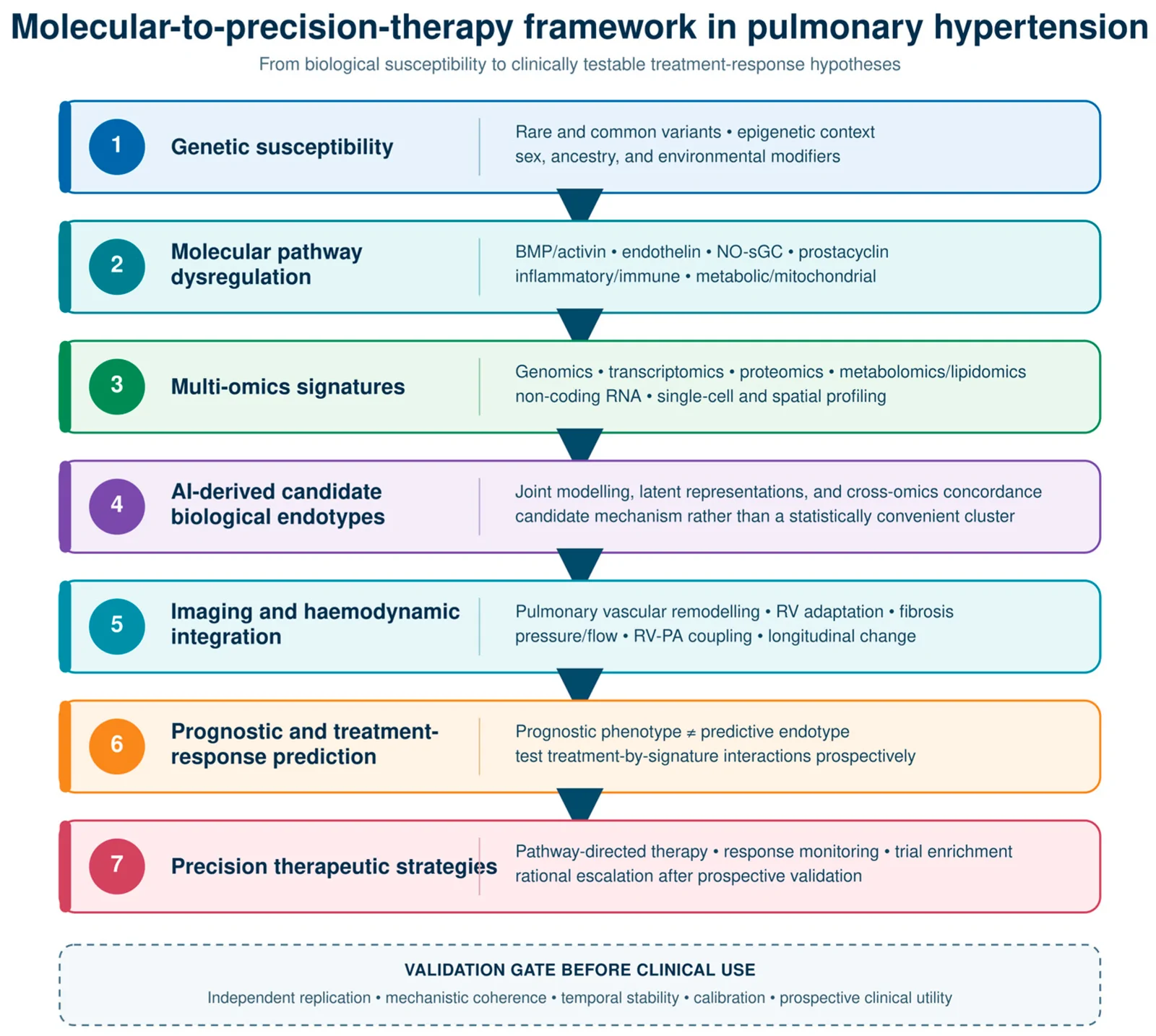
Molecular-to-precision-therapy framework in pulmonary hypertension. Genetic and epigenetic susceptibility converges on dysregulated vascular, immune, and metabolic pathways that generate measurable multi-omics signatures. AI can integrate these molecular data with imaging and haemodynamics to define candidate biological endotypes and estimate prognosis or treatment response. Translation to precision therapy requires independent replication, mechanistic coherence, temporal stability, calibration, and prospective demonstration of clinical utility and treatment-effect modification; candidate endotypes are therefore not current prescribing rules. This figure was created with the assistance of artificial intelligence and subsequently reviewed and curated by the authors. The molecular evidence underpinning this framework is strongest in PAH; extension to other PH groups remains investigational and requires disease-specific validation.

The central molecular-to-precision-therapy argument is summarised in Figure 3. Genetic susceptibility and acquired molecular dysregulation generate multi-omics signatures that AI can integrate with organ-level imaging and haemodynamic phenotypes. The resulting subgroup should be regarded as a candidate biological endotype until it satisfies the validation criteria defined above, only then can it support treatment-response hypotheses, trial enrichment, or precision therapeutic strategies.

## 8. Cross-Cutting Methodological, Implementation, and Regulatory Standards

Despite promising diagnostic and prognostic performance, translation of AI into routine PH care remains constrained by methodological, regulatory, and infrastructural barriers. The published literature is dominated by retrospective studies from selected expert centres, often with limited sample size, class imbalance, incomplete reporting, and insufficient external validation [18,19]. Models trained in a narrow referral population may fail when applied to community cohorts, different PH groups, new equipment, altered coding systems, or evolving treatment pathways. Development and evaluation must therefore anticipate dataset shift and establish transportability rather than relying on random internal splits.

Explainability is particularly important when predictions influence invasive testing, treatment escalation, or transplantation referral. Complex neural networks and ensemble models may be difficult to interrogate directly, but explainable-AI methods can estimate the contribution of individual variables or spatial regions to a prediction. Feature-importance and attribution estimates describe model behaviour rather than causal effects; they should therefore be treated as model diagnostics, not as evidence that an attributed variable or image region is mechanistically involved in PH. They require clinical plausibility testing, stability analysis, and assessment of whether they improve clinician understanding without producing false reassurance. Visual localisation in imaging models and feature-attribution methods for clinical models may support this objective, but should complement rather than replace transparent model design and uncertainty reporting [19,23].

Regulatory evaluation should distinguish automated measurement from autonomous diagnosis and adaptive decision support. AI-enabled echocardiographic software can standardise extraction of variables such as tricuspid regurgitation velocity, right atrial area, and RV dimensions, and has demonstrated agreement with expert measurements in PH cohorts [29,30]. Nevertheless, regulatory clearance for a technical function does not establish clinical utility across all PH populations. Post-deployment surveillance, version control, monitoring for performance drift, and clear assignment of professional accountability are required.

Methodological and reporting standards provide a minimum framework for trustworthy evidence. TRIPOD+AI requires transparent description of data sources, participants, outcomes, missing data, model development, hyperparameter tuning, performance, calibration, and fairness [89]. Interventional studies of AI-assisted care should follow CONSORT-AI, while protocols should follow SPIRIT-AI [90,91]. Risk-of-bias assessment, prespecified analysis plans, external validation, and clinically meaningful comparators are essential, particularly when the number of candidate predictors is large relative to the number of events.

Data fragmentation is a structural obstacle in a rare and heterogeneous disease. Harmonised multicentre registries should combine standardised clinical data with electrocardiography, imaging, invasive haemodynamics, biospecimens, treatment trajectories, and longitudinal outcomes. PVDOMICS illustrates the value of deep phenotyping across conventional diagnostic groups and provides a template for linking high-dimensional molecular data to clinically meaningful phenotypes [92]. Federated learning may extend multicentre development by allowing institutions to train shared models without central transfer of raw patient-level data [93]. It does not, however, eliminate differences in acquisition, coding, case mix, or governance, and model updates may themselves create privacy risks. Interoperable data models, harmonised acquisition protocols, robust cybersecurity, predefined validation plans, and independent auditing are therefore cross-cutting prerequisites rather than future-model-specific considerations.

For formal appraisal of prediction-model studies, PROBAST+AI provides an established framework for assessing model-development quality, risk of bias, and applicability [94]. We did not retrospectively assign PROBAST+AI ratings to the prediction-model reports selected for this review because the manuscript was designed as a broad narrative synthesis spanning prediction, clustering, imaging automation, omics, mechanistic, and interventional evidence rather than as a systematic review of a complete prediction-model corpus. Applying a formal tool only to a purposively selected subset would risk implying a degree of exhaustiveness and comparability that the review design does not support. This is an explicit limitation: purposive selection is susceptible to selective citation, and the qualitative appraisal used here should not be interpreted as a formal risk-of-bias-weighted evidence synthesis. Future systematic updates focused on prediction models should prespecify eligibility criteria and apply PROBAST+AI across the complete eligible corpus.

## 9. Future Research Architectures: Multimodal Foundation Models and Digital Twins

Multimodal foundation models represent a second emerging direction. In principle, systems pretrained on large and diverse datasets could integrate free-text reports, serial electrocardiograms, echocardiography, cardiac magnetic resonance, computed tomography, right-heart catheterisation waveforms, circulating biomarkers, genomics, and other omics layers within a unified representation [95]. Such models could support tasks that are currently addressed separately, including diagnostic referral, phenotypic classification, outcome prediction, and identification of treatment-response signatures. For PH, however, scale alone will not ensure validity. Development must preserve disease-specific physiological constraints, explicitly represent missingness and measurement uncertainty, and undergo evaluation across PH groups, referral settings, demographic strata, and equipment vendors. Particular attention is required to prevent common phenotypes from obscuring rare but clinically consequential subgroups.

Digital twins are a more speculative extension of this multimodal architecture. A clinically meaningful PH digital twin would be a dynamic, patient-specific model repeatedly updated with haemodynamic, imaging, biomarker, functional, molecular, and treatment data. It would aim to simulate changes in right-ventricular–pulmonary-arterial coupling, congestion, exercise capacity, or projected risk under alternative therapeutic strategies [84,86,88,96]. Current healthcare digital twins are often static prediction models and do not demonstrate counterfactual validity; accordingly, no PH digital twin is ready for clinical decision-making [96].

These architectures introduce questions beyond the cross-cutting standards consolidated in Section 8. Multimodal foundation models will need to show that pretraining across heterogeneous data adds task-specific value without obscuring rare PH phenotypes, whereas digital twins require credible counterfactual validity: simulated responses to alternative interventions must correspond to observed responses when prospectively tested [84,86,88,95,96]. Until these additional requirements are met, both approaches should be regarded as research architectures rather than clinical decision systems.

## 10. Conclusions

Artificial intelligence offers a framework for integrating clinical, electrical, imaging, haemodynamic, biomarker, and molecular information across PH. The scope of evidence is task-dependent: screening, referral, and organ-level phenotyping studies span several PH groups, whereas validated risk algorithms, multi-omics endotyping, and pathway-directed therapeutic strategies are concentrated predominantly in PAH. Current models show promise for detection, phenotyping, and risk stratification, but most evidence remains retrospective, derives from selected referral populations, and lacks sufficient external or prospective validation for autonomous clinical use [15,16,17,18,19,23,25,26,27,28,29,30,31,32,33,34,35,36,83,84,86,87,88]. Predictive accuracy alone is therefore an insufficient translational endpoint. Clinically useful systems must be calibrated, robust across populations and settings, interpretable, and prospectively shown to improve clinically meaningful decisions and patient-centred outcomes [18,19,89,90,91,92,93,94].

The more consequential objective is not simply to build more accurate models, but to determine whether multimodal AI can help identify biologically validated and clinically actionable endotypes that connect molecular mechanisms with reproducible imaging, haemodynamic, biomarker, and clinical phenotypes and where relevant differential treatment response [17,24,81,84,86,88]. This molecular-to-therapeutic framework is currently supported most strongly in PAH, particularly idiopathic and heritable PAH, because comparable multi-omics and endotype-validation data remain sparse in other PH groups. Until such endotypes, their measurable surrogates, and treatment-by-signature interactions are prospectively validated, AI should remain a clinical decision-support tool that complements expert judgement rather than an autonomous diagnostic or therapeutic system [18,19,89,90,91,92,93,94,95].

## Figures and Tables

**Table 1:** Data modalities and AI tasks in pulmonary hypertension.

Data Source	Representative Inputs	AI Tasks and Potential Outputs	Principal Limitations
Clinical data and EHRs	Symptoms, diagnoses, medication exposure, healthcare use, laboratory trajectories	Case finding, referral prioritisation, missing-data-aware risk prediction, longitudinal phenotyping	Coding heterogeneity, informative missingness, temporal drift, confounding by healthcare access
Electrocardiography	Raw 12-lead waveforms, intervals, rhythm and morphology	Early detection, right-heart stress signatures, phenotyping of pre- versus post-capillary disease	Spectrum bias, device and site effects, limited mechanistic interpretability
Echocardiography	TRV, RV dimensions, TAPSE, FAC, strain, RA area, cine loops	Automated measurement, PH probability estimation, RV phenotyping and prognosis	Image quality, vendor dependence, absent TR signal, threshold uncertainty
Cardiac magnetic resonance	RV volumes, mass, ejection fraction, tissue features, three-dimensional motion	Automated segmentation, motion phenotyping, haemodynamic estimation and outcome prediction	Referral-centre datasets, acquisition heterogeneity, cost and availability
CT/CTPA	Vascular calibre and pruning, perfusion, thromboembolic lesions, parenchymal radiomics	Aetiological classification, CTEPH support, quantitative vascular and lung phenotyping	Radiomic instability, reconstruction dependence, radiation and contrast exposure
Right-heart catheterisation	Pressure and flow waveforms, mPAP, PAWP, PVR, RAP, cardiac index, SvO_2_	Physiological phenotyping, quality control, trajectory and outcome modelling	Invasive acquisition, waveform artefacts, centre-specific protocols
Biomarkers and multi-omics	Proteins, metabolites, RNA, genetic variants, single-cell and spatial data	Candidate molecular phenotype discovery, endotype validation, biomarker panels, target discovery and trial enrichment	High dimensionality, batch effects, limited sample size, uncertain causality
Wearables and remote monitoring	Heart rate, activity, oxygen saturation, symptoms and home measurements	Early deterioration alerts, treatment-response monitoring and adaptive follow-up	Adherence, missingness, device variability, alert burden and privacy

Note: Abbreviations: AI, artificial intelligence; CT, computed tomography; CTPA, computed tomography pulmonary angiography; CTEPH, chronic thromboembolic pulmonary hypertension; EHR, electronic health record; FAC, fractional area change; mPAP, mean pulmonary arterial pressure; PAWP, pulmonary arterial wedge pressure; PH, pulmonary hypertension; PVR, pulmonary vascular resistance; RA, right atrium; RAP, right atrial pressure; RNA, ribonucleic acid; RV, right ventricle; SvO_2_, mixed venous oxygen saturation; TAPSE, tricuspid annular plane systolic excursion; TR, tricuspid regurgitation; TRV, tricuspid regurgitation velocity.

**Table 2:** Evidence map of representative molecular and omics studies in pulmonary hypertension.

Study/Species-Population/PH Group	Tissue or Biospecimen	Sample Size/Platform	Endpoint or Biological Question	Validation Approach/Independent Replication	Main Limitation
Gräf et al. [60] | Human, multicentre PAH cohort (predominantly idiopathic; also familial and drug-associated PAH; small PVOD/PCH subset)	Blood/saliva DNA	1038 PAH index cases + 6385 non-PAH controls; Illumina WGS	Rare-variant enrichment and discovery of PAH susceptibility genes	Case–control analysis, familial segregation, and functional testing of selected variants; multicentre ascertainment, but no dedicated external cohort for all novel loci	Genetic susceptibility rather than active disease state; ancestry imbalance and incomplete penetrance limit direct endotyping or treatment inference
Courboulin et al. [64] | Human group 1 PAH plus mouse hypoxia and rat monocrotaline PH	Human lung and distal PASMCs; rodent lung	Human lung: 8 PAH + 8 controls; PASMC confirmation: 3 PAH + 5 controls; miRNA profiling/qRT-PCR plus mechanistic assays	miR-204 dysregulation, relation to disease severity, and proliferative/anti-apoptotic signalling	qRT-PCR confirmation, rodent disease models, pathway perturbation, and synthetic miR-204 rescue; no independent human replication cohort	Small explant-derived human samples and cross-species inference; mechanistic evidence is stronger than clinical generalisability
Rhodes et al. [67] | Human idiopathic/heritable PAH	Plasma	Development: 218 PAH (143 + 75); longitudinal cohort: 43; external validation: 93; aptamer assay of 1129 proteins with targeted-assay confirmation	All-cause mortality and incremental prognostic information beyond established clinical assessment	Independent Paris validation cohort, targeted protein confirmation, longitudinal paired sampling, and comparison with REVEAL	Observational prognostic study restricted to selected PAH phenotypes; does not establish causal endotypes or treatment-effect modification
Rhodes et al. [69] | Human idiopathic/heritable PAH with healthy and disease controls	Plasma	365 PAH + 121 healthy controls + 139 symptomatic disease controls; UPLC-MS metabolomics	Disease discrimination, survival associations, and treatment-associated longitudinal metabolic change	Multiple-testing correction, disease-control comparison, confounder adjustment, and longitudinal sampling; independent external metabolomic replication not reported	Systemic and pre-analytical confounding; observational associations do not establish pathway causality or predictive treatment biomarkers
Saygin et al. [71] | Human IPAH	Explanted lung tissue	3 IPAH + 6 control lungs; 10× Genomics scRNA-seq (~3688 cells/sample on average)	Cell-type composition and disease-associated transcriptional programmes in lung vasculopathy	Histopathological confirmation and cell-cluster/pathway analyses; no independent external cohort	Very small, end-stage transplant cohort with treatment exposure; tissue availability and disease-stage bias
Asosingh et al. [72] | Human PAH	PAEC isolated from explanted pulmonary arteries	3 PAH + 3 donor controls; 72,454 endothelial cells; 10× single-cell transcriptomics	Endothelial heterogeneity and proliferative/angiogenic transcriptional states	Within-dataset clustering and differential-expression/pathway analyses; no independent external donor cohort	Only six donors and selected cultured/isolated endothelial cells; cell-isolation and end-stage disease bias
Hemnes et al. [75] | Human HPAH plus transgenic Bmpr2 mouse models	Human RV tissue; mouse RV	Human RV microarray: 2 HPAH + 2 controls + 2 DCM; Affymetrix Gene 1.0, lipid assays; complementary Bmpr2 mouse models	RV fatty-acid oxidation defects, lipid deposition, and BMPR2-related maladaptation	Cross-species concordance, pulmonary-artery banding controls, and metformin perturbation in mice; no independent human RV replication cohort	Extremely small human tissue sample; mechanistic findings rely heavily on experimental models
Pi et al. [77] | Human mixed-aetiology PAH	Plasma	117 PAH; 7288 aptamers targeting 6467 proteins	Three-year mortality, RV dilation, NT-proBNP, and PVR-adjusted RV-vulnerability signatures	PLS-DA and multivariable/PVR-adjusted association models in the discovery cohort; findings confirmed in independent PAH cohorts using plasma proteomics and RV tissue gene- and protein-expression datasets	Single-centre discovery cohort with incomplete imaging/haemodynamic data for some endpoints; external confirmation was performed at protein/pathway level rather than through prospective validation of the complete prognostic models; associations are not treatment-predictive

Note: The table is intentionally study-level rather than technology-level. Sample sizes refer to the principal analytic cohorts reported in the primary studies, where a study combined human and experimental evidence; the human and model components are identified separately. “Independent replication” denotes validation in a distinct human cohort rather than technical confirmation alone. Abbreviations: BMPR2, bone morphogenetic protein receptor type 2; Bmpr2, murine gene symbol for BMPR2; DCM, dilated cardiomyopathy; DNA, deoxyribonucleic acid; HPAH, heritable pulmonary arterial hypertension; IPAH, idiopathic pulmonary arterial hypertension; miR-204, microRNA-204; miRNA, microRNA; NT-proBNP, N-terminal pro-B-type natriuretic peptide; PAEC, pulmonary artery endothelial cell; PAH, pulmonary arterial hypertension; PASMC, pulmonary artery smooth muscle cell; PCH, pulmonary capillary haemangiomatosis; PH, pulmonary hypertension; PLS-DA, partial least squares discriminant analysis; PVR, pulmonary vascular resistance; PVOD, pulmonary veno-occlusive disease; qRT-PCR, quantitative reverse transcription polymerase chain reaction; REVEAL, Registry to Evaluate Early and Long-term PAH Disease Management; RV, right ventricle; scRNA-seq, single-cell RNA sequencing; UPLC-MS, ultraperformance liquid chromatography–mass spectrometry; WGS, whole-genome sequencing.

**Table 3:** Completed integrative molecular and multimodal studies in pulmonary hypertension.

Study/PH Population	Integrated Molecular Layers	Clinical or Imaging Linkage	Integration Design	Validation	Principal Output and Current Limitation
Hong et al. [78] PAH lung biobank: 96 PAH, 52 controls	Bulk lung transcriptomics integrated with GWAS, Bayesian regulatory networks, single-cell transcriptomics, and pharmacotranscriptomics	Clinicopathological deep phenotyping; plasma assessment of ASPN in independent PAH cohorts	Cross-cohort systems integration centred on transcriptomic network modules; not all molecular layers measured jointly in the same participants	Independent PAH cohorts plus cellular perturbation and Sugen-hypoxia experiments	Prioritised ASPN as a potentially protective network hub. Strong mechanistic discovery, but not an externally validated diagnostic, prognostic, or treatment-response model.
Khassafi et al. [79] PH/PAH RV-remodelling cohorts	Human and experimental RV transcriptomics linked to circulating plasma proteomics	MRI-defined RV adaptation states, including RV–PA coupling; discovery plasma cohort included 35 PAH participants	Cross-tissue and cross-cohort molecular integration with imaging-defined phenotypes; not a single jointly trained within-participant multi-omics clinical model	Experimental models and independent clinical cohorts used for orthogonal confirmation	Identified extracellular-matrix signals associated with maladaptive RV remodelling. Translational biomarker evidence, but no prospectively validated endotype or treatment classifier.
Dan-zeng et al. [80] High-altitude PH: 30 HAPH, 30 matched controls	Within-participant plasma proteomics plus untargeted metabolomics	Clinical phenotyping; no integrated imaging model	Joint pathway-level multi-omics analysis	Targeted proteomic (PRM) confirmation of selected proteins; no independent external cohort	Identified coordinated lipid, immune, cytoskeletal, and oxidative-stress pathways. True molecular integration, but small single-centre discovery study without validated clinical endotyping.

Note: The table distinguishes within-participant multi-omics integration from cross-cohort systems integration and from molecular–imaging linkage. None of these studies establishes a clinically validated multimodal endotype or a treatment-response classifier. Abbreviations: ASPN, asporin; GWAS, genome-wide association study; HAPH, high-altitude pulmonary hypertension; MRI, magnetic resonance imaging; PAH, pulmonary arterial hypertension; PH, pulmonary hypertension; PRM, parallel reaction monitoring; RV, right ventricle; RV–PA coupling, right-ventricular–pulmonary-arterial coupling.

**Table 4:** Task-specific model performance in selected artificial-intelligence studies in pulmonary hypertension.

Study/Population	Intended Use; Inputs	Reference Outcome/Horizon	Sample Size; Events/Prevalence	Validation Design; Comparator	AUC (95% CI); Calibration	Threshold Metrics (Clinically Relevant Threshold When Reported)
Kiely et al. [15] iPAH case finding	Population screening; ≤5 y NHS healthcare-resource-use history, diagnoses, specialties, procedures, age	Expert-confirmed iPAH diagnosis; prediction from pre-diagnostic history up to 5 y	709 iPAH vs. 2,812,458 stratified controls; deployment prevalence modelled at 5.5/million	5-fold cross-validation only; no external validation; comparator: background prevalence/no model	AUC: NR; calibration: NR	At threshold selected to identify 100 true positives: sensitivity, 14.10%; specificity, 99.99%; PPV, 10.32%; NPV, 99.99%.
Dawes et al. [16] Newly diagnosed PH	Prognosis; 3D CMR RV motion plus conventional imaging, haemodynamic, functional, and clinical markers	1-y survival; median follow-up 4.0 y	n = 256; 93 deaths (36%) and 1 lung transplantation	Internal model development; no independent external cohort reported; comparator: conventional markers	AUC 0.73 vs. 0.60 for conventional markers (95% CI NR); calibration: NR	Sensitivity/specificity: NR; PPV/NPV: NR.
DuBrock et al. [25] Mixed PH-likely population	ECG screening; raw 12-lead ECG voltage–time data	PH-likely label by RHC or echocardiography; diagnostic window ±1 mo; pre-emptive 6–18 mo and up to 5 y	Mayo diagnostic test: 16,175 PH-likely/87,998 controls; VUMC: 6045/24,256. Clinical prevalences used for predictive values: 2.87% and 3.09%	Mayo train/validation/test split plus external VUMC validation; no conventional clinical model comparator	Mayo AUC, 0.92 (0.92–0.92); VUMC, 0.88 (0.87–0.88); calibration: NR	Threshold, 0.308. Mayo: sensitivity, 85.51%, specificity, 83.81%, PPV, 13%, NPV, 99% at 2.87% prevalence. VUMC: 82.33%, 76.95%, 10%, 99% at 3.09% prevalence.
Aras et al. [26] Overall PH and subtypes	ECG detection/subtyping; 12-lead ECG voltage data	PH by RHC or echocardiogram within 90 d; contemporaneous detection and ECGs up to 2 y before diagnosis	Overall cohort 5016 PH/19,454 controls; principal test set 982 PH/3903 controls (20% prevalence)	Single-centre 7:1:2 train/development/test split; no external site; comparator: reference haemodynamic/echo labels	Overall PH AUC, 0.89 (0.88–0.90); pre-capillary, 0.91 (0.89–0.94); PAH, 0.94 (0.92–0.96); group 3 PH, 0.90 (0.89–0.91); calibration: NR	Overall PH at 20% prevalence: sensitivity, 0.79 (0.76–0.81), specificity, 0.84 (0.83–0.85), PPV, 0.56 (0.53–0.58), NPV, 0.94 (0.93–0.95). At simulated 1% prevalence: PPV, 0.05; NPV, 1.00.
Kwon et al. [27] Mixed PH; echocardiographic label	ECG screening/future risk; 12-lead ECG plus demographics	Echocardiographic PH diagnosis; contemporaneous detection; secondary future echocardiographic PH during follow-up	Derivation, 24,202; internal validation, 3174; external validation, 10,865; 4096 PH among 38,241 overall (10.7%). Future-risk cohort n = 2939 baseline non-PH	Historical two-hospital derivation, internal and external validation; comparator: NR	AUC, 0.859 internal; 0.902 external (95% CI NR); calibration: NR	Sensitivity/specificity and PPV/NPV: NR. Future echocardiographic PH: 31.5% in AI high-risk vs. 5.9% low-risk.
Anand et al. [28] Suspected/mixed PH	RHC referral support; 19 clinical/echocardiographic features, not requiring TRV	RHC mPAP > 20 mmHg within 1 wk of echo; contemporaneous diagnosis	n = 7853; PH, 6323 (81%); test n = 1554 with 1242 PH (~80%)	Train n = 5024/validation n = 1275/test n = 1554; 5-fold CV in training; no external centre; comparator: no prespecified alternative model	Test AUC, 0.83 (0.80–0.85); calibration: NR	Sensitivity, 88%; specificity: NR; PPV, 89%; NPV, 54%.
Salehi et al. [29] Suspected PH (ASPIRE)	Automated echocardiographic measurement; AI-derived TRJV	RHC mPAP > 20 mmHg; contemporaneous diagnostic assessment	n = 1031; 771 PH (75%); automated TRJV measurable in 87%	Retrospective registry cohort; no independent external cohort; comparator: manual TRJV	Automated AUC, 0.88 (0.84–0.90) vs. manual, 0.88 (0.86–0.91); calibration: NR	Threshold-dependent sensitivity/specificity reported at TRJV 2.8 and 3.4 m/s and vary by haemodynamic definition; no single universal operating point. PPV/NPV: NR.
Celestin et al. [30] PAH case–control and mild-PH referral cohorts	Automated TRV measurement/detection; deep-learning echo analysis	RHC mPAP > 20 mmHg; mild haemodynamic range emphasised in referral cohort	Case–control: 221 PAH/213 healthy. Referral cohort n = 196; 107 mPAP 20–35 and 89 <20; TRV measurable in 171	Single-centre cohorts; comparator: clinical laboratory TRV interpretation	Mild-PH referral: DL AUC, 0.75 (0.67–0.81) vs. clinical, 0.79 (0.72–0.85); case–control AUC, 0.98 vs. 0.99; calibration: NR	Sensitivity/specificity: NR for principal mild-PH AUC comparison; PPV/NPV: NR.
Lui et al. [31] Systemic sclerosis	RHC referral support; PFT, ECG, echocardiography, CT	RHC-confirmed PH; contemporaneous diagnosis	n = 130; 66 PH (50.8%)	Internal train–test split; no external validation; comparators: CART and logistic regression	Random-forest AUC, 0.92 (0.83–1.00); calibration: NR	Sensitivity, 0.95 (0.75–1.00); specificity, 0.80 (0.56–0.94); PPV/NPV: NR.
Huang et al. [35] Mixed PH; chest radiography	Low-cost imaging screening; chest-radiograph deep-learning classifier (CXR-PH-Net)	PH detection; RHC-confirmed internal and external validation cohorts; contemporaneous classification	Overall retrospective cohort *n* = 4576 (2288 PH); RHC-confirmed internal cohort *n* = 1158; external RHC cohort *n* = 90; cohort-specific prevalence in the RHC validation sets: NR in primary abstract	Internal testing plus RHC-confirmed internal validation and external RHC validation from two independent hospitals; comparator: no guideline-based same-cohort comparator reported	RHC-confirmed internal AUC, 0.872; external RHC AUC, 0.811 (95% CIs NR in primary abstract); calibration: NR	Sensitivity, 0.902 in the RHC-confirmed internal cohort and 0.803 in the external RHC cohort; specificity and PPV/NPV for these RHC cohorts: NR in primary abstract
Guo et al. [36] Mixed screening population	Low-cost screening; digital-stethoscope phonocardiograms/mel-spectrograms	Echocardiographic estimated PASP ≥ 40 mmHg (surrogate, not RHC-confirmed PH); contemporaneous	Training: ~6000 labelled PCGs plus ~169,000 unlabelled PCGs; independent labelled test *n* = 196; test prevalence: NR	5-fold cross-validation plus held-out test set; comparator: NR	Mean CV AUC, 0.79 (fold range 0.76–0.85); calibration: NR	Held-out test: sensitivity, 0.71; specificity, 0.73; PPV/NPV: NR.
Liao et al. [40] Suspected PH	Automated echo probability; PSAX-PML image features	RHC-confirmed PH within 24 h of echo; contemporaneous diagnosis	*n* = 346; development/internal *n* = 275; external *n* = 71 (58 PH, 13 non-PH; 81.7% prevalence)	Two-centre development with external validation; comparator: traditional echocardiographic assessment	Development AUC, 0.945 vs. 0.892 comparator; external AUC, 0.950 (0.897–1.000); calibration: NR	Sensitivity/specificity: NR in principal report summary; PPV/NPV: NR.
Hirata et al. [41] Non-PH vs. pre-capillary vs. post-capillary PH	PH classification; 24 physical/echocardiographic parameters	RHC-based 3-class haemodynamic classification; contemporaneous	*n* = 885; derivation *n* = 720; prospective validation *n* = 165; class prevalence/event counts in validation: NR in abstract	Derivation plus prospective validation; comparator: guideline-based echocardiographic assessment	Derivation AUCs: non-PH, 0.789; pre-capillary PH, 0.766; post-capillary PH, 0.742 (95% CIs NR); calibration: NR	Macro-classification accuracy 59.4% vs. 51.6% guideline comparator in derivation; threshold-specific sensitivity/specificity and PPV/NPV: NR.
Kanwar et al./PHORA [83] PAH	1-y risk stratification; REVEAL 2.0 clinical, functional, and haemodynamic variables in a tree-augmented naïve Bayesian network	1-y survival/12-mo mortality risk	REVEAL cohort size: NR in the primary performance summary used for extraction; external COMPERA *n* = 3849 and PHSANZ *n* = 978; 1-y event counts/prevalence: NR	REVEAL model development + 10-fold cross-validation; independent external COMPERA/PHSANZ validation. Same-cohort comparator: REVEAL 2.0 in REVEAL	REVEAL AUC, 0.80 vs. REVEAL 2.0, 0.76; COMPERA, 0.74; PHSANZ, 0.80. 95% CIs: NR/not uniform. Formal calibration slope/intercept/Brier score: NR; risk-category survival separation reported	Single-threshold sensitivity/specificity and PPV/NPV: NR; prespecified 12-mo mortality-risk categories used.

Note: Metrics are reproduced in the context of the original study target and should not be compared across rows as if they represented a common estimand. No unreported confidence interval, calibration statistic, event count, prevalence, or predictive value was imputed or back-calculated. “NR” indicates not reported in the primary publication or primary report summary available for extraction. Abbreviations: 3D, three-dimensional; AI, artificial intelligence; ASPIRE, Assessing the Spectrum of Pulmonary Hypertension Identified at a Referral Centre; AUC, area under the receiver operating characteristic curve; CART, classification and regression tree; CI, confidence interval; CMR, cardiac magnetic resonance; COMPERA, Comparative, Prospective Registry of Newly Initiated Therapies for Pulmonary Hypertension; CT, computed tomography; CV, cross-validation; CXR-PH-Net, chest-radiograph-based pulmonary hypertension network; d, day(s); DL, deep learning; ECG, electrocardiography; h, hour(s); iPAH, idiopathic pulmonary arterial hypertension; m/s, metres per second; mmHg, millimetres of mercury; mo, month(s); mPAP, mean pulmonary arterial pressure; NHS, National Health Service; NPV, negative predictive value; NR, not reported; PAH, pulmonary arterial hypertension; PASP, pulmonary artery systolic pressure; PCG, phonocardiogram; PFT, pulmonary function testing; PH, pulmonary hypertension; PHORA, Pulmonary Hypertension Outcomes Risk Assessment; PHSANZ, Pulmonary Hypertension Society of Australia and New Zealand; PPV, positive predictive value; PSAX-PML, parasternal short-axis view at the papillary-muscle level; REVEAL, Registry to Evaluate Early and Long-term PAH Disease Management; RHC, right-heart catheterisation; RV, right ventricle; TRV/TRJV, tricuspid regurgitation velocity/jet velocity; VUMC, Vanderbilt University Medical Centre; wk, week(s); y, year(s). For studies with multiple validation datasets, each AUC is tied to its corresponding reference label and validation cohort rather than presented as a single model-wide performance estimate. In the PHORA study, COMPERA and PHSANZ served as independent external registries. Where the primary performance summary did not provide a uniform confidence interval, event count, or formal calibration statistic, the field is reported as NR rather than reconstructed.

**Table 5:** Molecular pathways, candidate endotypes, and therapeutic implications in pulmonary arterial hypertension.

Molecular Pathway	Measurable Biomarker or Omics Signature	Candidate AI-Defined Endotype → Therapeutic Implication	Current Evidence Status
BMPR2/TGF-β/activin signalling	Pathogenic BMPR2 or related variants; relative BMP-SMAD1/5/9 and activin/TGF-β-SMAD2/3 activity; circulating activin-family proteins; pathway transcriptomic or proteomic signatures [59,60,61,62,63].	BMP-deficient or activin-dominant vascular-proliferative endotype → rationale for activin-ligand trapping with sotatercept and possible future trial enrichment; genotype or pathway status is not a validated treatment-selection marker [63,81,85].	Established clinical application of pathway-directed therapy; endotype-guided selection unproven.
Endothelin signalling	Circulating endothelin-1; EDN1, EDNRA, and EDNRB expression; endothelial transcriptomic or proteomic modules [5,86,88].	Endothelin-dominant vasoconstrictive or proliferative endotype → possible differential sensitivity to endothelin-receptor antagonists; no predictive signature has been validated.	Established clinical application; biomarker- or AI-guided selection investigational.
Nitric oxide-sGC signalling	Nitric-oxide metabolites, cyclic guanosine monophosphate, NOS3 or sGC-pathway expression, and endothelial-dysfunction signatures [5,86,88].	Nitric-oxide-deficient or sGC-impaired endotype → potential differential response to phosphodiesterase type-5 inhibition or sGC stimulation; not validated for drug choice.	Established clinical application; biomarker- or AI-guided selection investigational.
Prostacyclin signalling	Prostacyclin metabolites; PTGIS or prostacyclin-receptor expression; platelet or endothelial transcriptomic and proteomic signatures [5,86,88].	Prostacyclin-deficient endotype → possible prediction of prostacyclin-pathway response or need for escalation; no molecular classifier has been validated.	Established clinical application; biomarker- or AI-guided selection investigational.
Inflammatory and immune pathways	Cytokine panels; immune-protein clusters; circulating-leukocyte transcriptomics; spatial or single-cell immune states [17,24,65,71,74,78].	Immune-high or pathway-specific inflammatory endotype → trial enrichment for immunomodulatory or repurposed therapies; treatment-by-endotype interactions remain unproven.	Emerging association; therapeutic application investigational.
Metabolic and mitochondrial pathways	Metabolomic or lipidomic profiles; glycolytic and fatty-acid-oxidation programmes; miR-124/PKM2 signals; right-ventricular lipotoxicity markers [66,69,70,75,76,77,78,79,80].	Glycolytic, mitochondrial, or lipotoxic endotype → rationale for metabolic or mitochondrial interventions and longitudinal pharmacodynamic monitoring.	Emerging association to investigational hypothesis; no validated endotype-guided therapy.

Note: Candidate AI-defined endotypes and their therapeutic implications are hypothesis-generating and should not be interpreted as validated treatment-selection rules. Abbreviations: AI, artificial intelligence; BMP, bone morphogenetic protein; BMPR2, bone morphogenetic protein receptor type 2; EDN1, endothelin 1; EDNRA, endothelin receptor type A; EDNRB, endothelin receptor type B; miR-124, microRNA-124; NOS3, nitric oxide synthase 3; PKM2, pyruvate kinase M2; PTGIS, prostaglandin I_2_ synthase; sGC, soluble guanylate cyclase; SMAD1/5/9, SMAD family members 1, 5, and 9; SMAD2/3, SMAD family members 2 and 3; TGF-β, transforming growth factor beta.

**Table 6:** Pulmonary hypertension group, clinical task, predominant data modalities, and current evidence maturity.

PH Group	Principal Clinical Task	Predominant Data Modalities	Current Evidence Maturity
Group 1: PAH (molecular evidence strongest in idiopathic and heritable PAH)	Early detection and referral; haemodynamic classification; validated PAH risk stratification; molecular phenotype/endotype discovery; treatment-response hypotheses and trial enrichment.	Clinical/EHR data, ECG, echocardiography, CMR, CT, right-heart catheterisation, circulating biomarkers, genomics, transcriptomics, proteomics, metabolomics and other omics.	Strongest and broadest evidence base. Guideline-based risk tools and pathway-directed therapies are established in PAH; AI-guided or molecular-endotype-guided drug selection remains investigational.
Group 2: PH associated with left-heart disease	Differentiate post-capillary PH and mixed phenotypes from pre-capillary/PAH presentations; cardiovascular phenotyping and prognostic characterisation.	Clinical/EHR data, echocardiography, CMR, right-heart catheterisation and selected CT features.	Emerging direct AI evidence for classification and phenotyping. Molecular-endotyping- and AI-guided therapeutic stratification are insufficiently validated; PAH-derived treatment rules should not be extrapolated.
Group 3: PH associated with lung disease and/or hypoxia	Detection in chronic lung disease; vascular-parenchymal phenotyping; severity assessment and referral.	Pulmonary-function data, CT/radiomics, echocardiography, clinical variables and right-heart catheterisation.	Emerging imaging- and clinical-AI evidence. Molecular endotyping and treatment-response prediction remain limited and disease-specific; PAH-derived molecular or drug-selection frameworks are not directly transferable.
Group 4: pulmonary artery obstruction, principally CTEPH	Detection and aetiological classification; anatomical and haemodynamic phenotyping; support for referral to expert surgical/interventional assessment.	CT pulmonary angiography, perfusion imaging, echocardiography, radiomic/vascular features and right-heart catheterisation.	Direct AI evidence is emerging mainly for imaging-based detection and classification. Definitive therapeutic decisions remain based on established expert anatomical and haemodynamic assessment; molecular AI endotyping is sparse.
Group 5: PH with unclear and/or multifactorial mechanisms	Disease-specific aetiological classification, phenotyping and risk characterisation.	Clinical data, disease-specific imaging, right-heart catheterisation and selected biomarkers.	Sparse and heterogeneous evidence. No generalisable AI-defined molecular endotype or therapeutic-stratification framework is established; conclusions require disorder-specific validation.

Note: Evidence maturity is qualitative and reflects the literature synthesised in this narrative review rather than a formal GRADE assessment. Abbreviations: AI, artificial intelligence; CMR, cardiac magnetic resonance; CT, computed tomography; CTEPH, chronic thromboembolic pulmonary hypertension; ECG, electrocardiography; EHR, electronic health record; GRADE, Grading of Recommendations Assessment, Development and Evaluation; PAH, pulmonary arterial hypertension; PH, pulmonary hypertension.

## Data Availability

No new data were created or analysed in this study. Data sharing is not applicable to this article.
